# Implication of Stm1 in the protection of eIF5A, eEF2 and tRNA through dormant ribosomes

**DOI:** 10.3389/fmolb.2024.1395220

**Published:** 2024-04-18

**Authors:** Mengtan Du, Xin Li, Wanlin Dong, Fuxing Zeng

**Affiliations:** ^1^ Department of Systems Biology, School of Life Sciences, Southern University of Science and Technology, Shenzhen, China; ^2^ Institute for Biological Electron Microscopy, Southern University of Science and Technology, Shenzhen, China

**Keywords:** stm1, SERBP1, dormant ribosome, cryo-EM, eEF2

## Abstract

**Background:** Dormant ribosomes are typically associated with preservation factors to protect themselves from degradation under stress conditions. Stm1/SERBP1 is one such protein that anchors the 40S and 60S subunits together. Several proteins and tRNAs bind to this complex as well, yet the molecular mechanisms remain unclear.

**Methods:** Here, we reported the cryo-EM structures of five newly identified Stm1/SERBP1-bound ribosomes.

**Results:** These structures highlighted that eIF5A, eEF2, and tRNA might bind to dormant ribosomes under stress to avoid their own degradation, thus facilitating protein synthesis upon the restoration of growth conditions. In addition, Ribo-seq data analysis reflected the upregulation of nutrient, metabolism, and external-stimulus-related pathways in the *∆stm1* strain, suggesting possible regulatory roles of Stm1.

**Discussion:** The knowledge generated from the present work will facilitate in better understanding the molecular mechanism of dormant ribosomes.

## 1 Introduction

In a typical human cell, ribosomes are present in a quantity of around 10^7^ per cell, and ribosomal proteins constitute approximately 4%–6% of the total protein mass (Shore and Albert, 2022). The ribosome pool undergoes meticulous regulation to ensure appropriate translational capacity. Under nutrient-rich conditions, cells enhance ribosome biogenesis to facilitate protein synthesis. In contrast, cells reduce ribosome biogenesis and employ mechanisms such as autophagy (ribophagy) and/or proteasomal degradation to degrade ribosomes in response to nutrient-deprived conditions (Kraft et al., 2008; Wang et al., 2020). However, excessive degradation may deplete the ribosomal reserves and impede the resumption of cell growth upon the restoration of favorable growth conditions. To mitigate this risk, certain factors, known as preservation factors, safeguard a small population of non-translating, vacant ribosomes (Van Dyke et al., 2006; Balagopal and Parker, 2011; Prossliner et al., 2018; Wells et al., 2020). The precise mechanisms governing the preservation and subsequent reactivation of mRNA-free dormant ribosomes still remain enigmatic (Baudin et al., 2021; Kisonaite et al., 2022; Shetty et al., 2023a; Leesch et al., 2023).

Translationally inactive ribosomes have been observed in both prokaryotes and eukaryotes. In response to stress stimuli, prokaryotic 70S ribosomes dimerize to form 100S particles via the association of hibernation-promoting factor (HPF) and ribosome modulation factor (RMF) in Gram-negative bacteria, or long-form hibernation-promoting factor (LHPF) in Gram-positive bacteria (Beckert et al., 2018). In eukaryotes, several factors, including SERBP1 (Stm1 in yeast), IFRD2, and CCDC124 (Lso2 in yeast), are associated with dormant ribosomes upon sporulation (MDF1 and MDF2 in microsporidia) and nutrient deprivation (Brown et al., 2018; Wells et al., 2020). In egg cells, dormant ribosomes are associated with four conserved factors, including Habp4, eEF2, Dap1b/Dap, and eIF5A (Leesch et al., 2023). Among these, eEF2 (eukaryotic elongation factor 2) and eIF5A (eukaryotic translation initiation factor 5A) are integral components of translation factors that are indispensable for mRNA translation regardless of the extent of translation homeostasis (Yuan et al., 2023). eEF2 mediates ribosomal translocation and transiently interacts with the tRNA-mRNA complex at the aminoacyl (A) site of the ribosome (Flis et al., 2018). While, eIF5A usually promotes translation elongation and termination upon ribosome stalling (Schuller et al., 2017). Dormant ribosomes offer a mechanism to exclude ribosomes from translation while also protecting them from degradation (Smith et al., 2022). To gain structural insights into the translational regulatory mechanism, the relationship between dormant ribosomes and their corresponding ribosome-bound translation factors remains an interesting scientific question for researchers.

Stm1, one of the yeast ribosome preservation factors, occupies the mRNA tunnel of the 40S subunit and inhibits translation by excluding the mRNA binding (Ben-Shem et al., 2011a). Similar to this, the non-structural protein 1 (Nsp1) of SARS-CoV-2 mediates translation inhibition by binding to the empty ribosome and blocking the mRNA channel (Thoms et al., 2020). Stm1 was initially shown to interact with translating ribosomes (Van Dyke et al., 2006). However, this interaction might be unspecific since a low KCl concentration (150 mM) could abolish the association of Stm1 with polysome (Shetty et al., 2023a). Additionally, almost no difference in the overall polysome profiles of wild-type and ∆*stm1* cells grown in nutrient-rich conditions. In contrast, ∆*stm1* showed a shift from 80S ribosomes to 40S and 60S subunits upon nitrogen starvation (Shetty et al., 2023a). Furthermore, cells lacking Stm1 are hypersensitive to nitrogen starvation or rapamycin treatment, suggesting a functional but so far poorly characterized interaction between Stm1 and TORC1 (Target of rapamycin complex 1). It was reported that TORC1 is an evolutionarily conserved serine/threonine kinase that can promote biogenesis and inhibit the degradation of ribosomes in response to nutrient availability by directly targeting Stm1/SERBP1 (Shetty et al., 2023b). In addition, Stm1 was also found to be associated with the 80S containing Reh1, a ribosome assembly factor that binds to pre-60S subunits at a late stage during their cytoplasmic maturation (Musalgaonkar et al., 2023). Besides its functional role in binding dormant ribosomes, yeast Stm1 has also been reported to bind G4 DNA or G4 RNA through a C-terminal RGG box-independent mechanism (Yan et al., 2021). In contrast, the human SERBP1 protein binds G-rich RNA through its C-terminal RGG box and can also undergo phase separation to regulate certain cellular processes (Baudin et al., 2021). Thus, it is evident that Stm1/SERBP1 plays diverse roles in the cell, necessitating further research to elucidate its specific pathways and mechanisms.

Here, we determined cryo-electron microscopy (cryo-EM) structures of five dormant ribosomes from yeast and human cells. Compared with the existing Stm1/SERBP1-containing ribosomes, some of our structures exhibit distinctly different small subunit rotations, providing more comprehensive structural insights into the state of dormant ribosomes. On the other hand, we observed structural conformational change in eEF2 in the dormant ribosomes, as compared to previous eEF2/ribosome complexes. Its domain III displayed considerable flexibility, to the extent that its electron density was not discernible. Based on the structural analysis of all Stm1/SERBP1-containing ribosomes, we postulated that eIF5A, E-tRNA, P/E-tRNA, Z-tRNA, pe/E-tRNA, and eEF2 can only transiently associate with the dormant ribosomes to avoid degradation, with no significant functional relevance to dormant ribosomes. Consistent with the speculation, 80S•Stm1 from monosome peak emphasize the presence of only Stm1 in dormant ribosomes obtained from highly active cells. In addition, Ribo-seq data analysis of ∆*stm1* revealed a moderate influence on metabolic, nutrient, and external stimuli-related pathways.

## 2 Materials and methods

### 2.1 Isolation and structural analysis of human dormant ribosomes

Numerous particles for dormant ribosomes were isolated from human cells utilizing affinity purification protocol, as previously described (Brown et al., 2018). This was also the case for three of the five dormant ribosome complexes reported in this study, including SERBP1•eEF2•eIF5A and SERBP1•eEF2•E-tRNA from human and 80S•Stm1•eIF5A from yeast.

The two human dormant ribosome complexes, SERBP1•eEF2•eIF5A and SERBP1•eEF2•E-tRNA, were isolated from datasets collected for pseudoknot/ribosome complexes by Relion. Briefly, HEK293F cells (ATCC CRL-1573) were grown in Union293 medium (UnonBotech) until 2 × 10^6^ cell⋅mL^−1^ and harvested by centrifugation at 3,000 rpm, 4°C for 20 min. Cell extracts were prepared using dounce tissue grinder. The ribosome particles were obtained via *in vitro* translation with these cell extracts. In this procedure, the *in vitro* translation was performed in buffer A (20 mM HEPES, pH 7.3, 120 mM KOAc, 2 mM Mg(OAc)_2_, 0.75 mM ATP, 0.1 mM GTP, 25 mM Creatine Phosphate, 1.67 mM DTT, 0.2 mg/mL Creatine Phosphokinase, 0.03 mg/mL amino acids mixture) and terminated with 100 μg/mL cycloheximide, followed by passing through Strep-avidin Magnetic Beads (Beyotime). For cryo-EM sample preparation, an aliquot of 2.5 µL of ribosomal complexes was applied onto the glow-discharged (15 mA for 30–45 s) holey carbon grids (quantifoil R1.2/1.3, Cu 300 mesh) with 2–4 nm continuous carbon film on top. After 30 s of incubation, grids were blotted for 3 s and plunged into liquid ethane using a Vitrobot device (FEI) operated at 4°C and 100% humidity. Cryo-grids were loaded onto Titan Krios electron microscope (300 keV, Thermo Fisher) equipped with Gatan K3 summit camera for data collection. Gain-normalized movies of 30 frames were collected with a total dose of 30 e^−^/pix. Image processing, including motion correction, contrast transfer function (CTF) estimation, particle picking, 2D and 3D classification, and final reconstruction were all performed in Relion 3.1 (Zivanov et al., 2018), utilizing MotionCorr2 (Zheng et al., 2017) and CTFFIND 4.1 (Rohou and Grigorieff, 2015). More or less the same procedures for cryo-EM data collections and processing throughout the study.

A total of 2854 movies were acquired, and 680,276 particles were initially picked using a Laplacian-of-Gaussian blob detection with diameters from 200 to 300 Å. After several rounds of 2D classification to remove the non-ribosomal particles, well-featured ribosome particles (227, 297) were selected for 3D classification, and 169, 732 particles were ribosomes with good reconstruction. A sphere mask on the GTPase-associated center (GAC) of the ribosome was used to isolate eEF2-bound ribosomes. Two classes with different 40S body rotations were obtained for the dormant ribosomes. Further classification with a sphere mask on the E-site of the ribosome was performed to remove the empty E-site particles, which reflected almost no ribosomes with an empty E-site at the end. Final reconstructions with a resolution of 3.04 Å and 2.98 Å were obtained for SERBP1•eEF2•eIF5A and SERBP1•eEF2•E-tRNA, respectively. Resolutions were reported based upon the gold-standard Fourier shell correlation (FSC) of 0.143 criterion.

### 2.2 Isolation and characterization of yeast dormant ribosomes bound with eIF5A

Similar to the two human dormant ribosome complexes, yeast 80S•Stm1•eIF5A ribosomal complex was obtained from *S.cerevisiae* cells (BY4742) in an exponential growth phase in the YPED medium (Zeng et al., 2021). Briefly, *S.cerevisiae* cells were grown to OD_600_ of 0.65 at 30°C, and treated with 100 mg/mL of cycloheximide for 2 min prior to harvesting at 3,000 rpm, 4°C for 10 min. Cells were resuspended in pre-cooled buffer B (20 mM HEPES-KOH, pH 7.4, 100 mM KOAc, 2.5 mM Mg(OAc)_2_, 1 mg/mL Heparin, 2 mM DTT, 0.5 mM PMSF). Cells were then grounded in liquid nitrogen to make the cell extracts for cryo-EM samples. Following cryo-EM data collection and processing with Relion, 80,383 particles were identified to bind with Stm1 and eIF5A, generating a reconstruction with an overall resolution of 3.59 Å.

### 2.3 Isolation of yeast dormant ribosomes bound with P/E-tRNA from crude ribosomes

For this dataset, the crude yeast 80S was studied and purified as described previously with minor modifications (Fernandez et al., 2013). Yeast cells (BY4742) were first grown and harvested as described above, but without adding CHX and ground in liquid nitrogen with precooled buffer B in 5 min. Cell lysates were clarified by 14,000 rpm, 4°C for 10 min and passed through 1 M sucrose cushion with buffer C (20 mM HEPES•KOH, pH 7.4, 100 mM KOAc, 2.5 mM Mg(OAc)_2_, 500 mM KCl, 2 mM DTT). Pellet was then resuspended in buffer B, followed by cryo-EM sample preparation. After several rounds of 2D and 3D classification, 421,211 particles were identified as ribosomal particles. Further classification removed the particles for subunits, and these showed severe orientation preferences. A final total of 199,598 particles were used for the reconstruction. The obtained complex was 80S•Stm1•P/E-tRNA with a resolution of 3.29 Å.

### 2.4 Monosome collection and structure determination

To characterize the structural details of monosomes, the yeast BY4742 strain was first grown in YPED medium at 30°C until OD_600_ was 0.6 in the exponential growth phase. A final concentration of 100 μg/mL of cycloheximide was supplemented to the cells and further incubated for 2 min. Cells were quickly collected at 30°C (3,000 rpm for 1 min with the supplemented CHX) and transferred into liquid nitrogen immediately. The collection and transfer were done in 2 min to prevent the cells from entering the stressful environment. Buffer B, containing 100 μg/mL of cycloheximide, was supplemented to the liquid nitrogen, followed by cell disruption with the tissue-crushing apparatus. After thawing, the cell extracts were obtained with centrifugation at 14,000 rpm, 4°C for 10 min, and 10 units of A_260_ were layered on top of 10%–50% sucrose gradient in buffer A containing the same concentration of cycloheximide. Ultracentrifugation was performed in SW 40 Ti rotor (Beckman) with a speed of 38,000 rpm for 3 h and 45 min at 4°C. Fractions were then collected using a homemade gradient collector, and the absorbance of ribosomes were monitored at 260 nm. Monosome fractions were pooled, and the ribosomes were pelleted down in TLA-100 rotor (Beckman) at 100,000 rpm for 20 min. Pellets were resuspended in buffer A and diluted to A_260_⋅of 4 for cryo-EM.

A total of 4,058 movies were acquired, and 971,190 particles were initially picked. After 2D classification, 630,862 particles were selected for further 3D classification, and 71% of particles were identified as good “empty 80S particles.” The final refinement of all these particles yielded a reconstruction of 80S ribosome bound Stm1 at a resolution of 2.88 Å. This complex was then assigned as 80S•Stm1.

### 2.5 Model building

For the two human (SERBP1•eEF2•eIF5A and SERBP1•eEF2•E-tRNA) and yeast (80S•Stm1•eIF5A) dormant ribosome complexes, coordinates were first rigid-body fitted into the density map. For human ribosomes, coordinates for 80S ribosome, eEF2, E-tRNA, and SERBP1 were all extracted from the available model of hibernating ribosomes (PDB: 6Z6M) (Wells et al., 2020). The AlphaFold predicted model for human eIF5A was used. For yeast ribosomes, coordinates of 80S ribosome and eIF5A were extracted from the Rbg1/Tma46-bound ribosomal complex (PDB: 7RR5) (Zeng et al., 2021), and Stm1 and P/E-tRNA were extracted from the empty 80S (PDB: 4V88) (Ben-Shem et al., 2011a) and *C. thermophilum* 80S ribosomes (PDB: 7OLD) (Kisonaite et al., 2022), respectively. Rigid-body fitting was carried out in UCSF Chimera (Pettersen et al., 2004) and Coot (Emsley et al., 2010). For 40S, the head and body were fitted separately. Similarly, each domain for eIF5A and eEF2 was also fitted separately. Stm1 and SERBP1 were fitted for each residues, and those with no obvious electron density were deleted in Coot. Refinement was carried out in Phenix (Adams et al., 2010).

### 2.6 Measurement of rotations

The 40S subunit rotation and head swiveling were measured in UCSF ChimeraX (Pettersen et al., 2021) utilizing “Match Maker” and command “measure rotation.” For the 40S body, the structures were aligned on large subunit rRNA, and the rotation between a pair of 40S body rRNAs was measured. For the 40S head, the structures were aligned on the 40S body rRNA, and the rotation between a pair of 40S head rRNAs was measured. Figures were prepared in UCSF Chimera (Pettersen et al., 2004) and UCSF ChimeraX (Pettersen et al., 2021).

### 2.7 Sequencing data sources

We retrieved eight sets of each RNA-Seq and Ribo-Seq datasets (in Sequence Read Archive, SRA format) from the Gene Expression Omnibus (GEO) database (BioProject Accession: PRJNA769126, or GEO: GSE185458). Supplementary Table S2 summarizes information about the sequencing data sources.

### 2.8 Pre-processing of raw data and analysis

The SRAToolkit was used to convert SRA to the FASTQ format. Subsequently, FastQC (Andrews, 2010) was utilized to assess the quality of each file, ensuring its suitability for analysis. Then, the raw reads were trimmed using Cutadapt (Martin, 2011) with specific parameters for RNA-Seq (a: AGA​TCG​GAA​GAG​CAC​ACG​TCT​GAA​CTC​CAG​TCA​C; minimum length: 20; q: 20) and Ribo-Seq samples (a: CTG​TAG​GCA​CCA​TCA​ATA​GAT​CGG​AAG​AGC​ACA​CGT​CTG​AAC​TCC​AGT​CAC; minimum length: 20; q: 20). Afterwards, the clean reads were aligned against ncRNA sequence set of *S*.*cerevisiae* obtained from NCBI using Bowtie2 (Langmead and Salzberg, 2012). Only unmapped non-ncRNA reads were considered for further analysis. Read mapping and counting against the *S.cerevisiae* S288C genome assembly (R64) (Engel et al., 2022) was performed with HISAT2 (Kim et al., 2019). The number of reads within the open reading frame of encoding genes were calculated with SAMtools (Li et al., 2009) and featurecounts.R program (Liao et al., 2019) to obtain the FPKM and TPM values. The pre-processed data were categorized into WT and ∆*stm1* based on experimental conditions. Translation efficiency was analyzed using Xtail software (Xiao et al., 2016). Differential genes in translation efficiency were further classified into upregulated and downregulated groups. Subsequently, GO pathway enrichment analysis for each group was performed with the Metascape online server (https://metascape.org). Finally, Ribotish (Zhang et al., 2017) was employed for quality control of Ribo-seq bam data. The periodicity of Ribo-seq dataset was calculated and visualized to illustrate frame bias and estimate P-site offset for different lengths of reads.

## 3 Results

Numerous studies reported the structures of dormant ribosomes bound with Stm1/SERBP1 from different species, including humans (Anger et al., 2013; Wells et al., 2020), rabbits (Brown et al., 2018; Leesch et al., 2023), mice (Smith et al., 2021a), *drosophila* (Anger et al., 2013), yeast (Ben-Shem et al., 2011a; Zhao et al., 2022), and *C. thermophilum* (Kisonaite et al., 2022). The main approach to acquiring them was to analyze the purified ribosomes obtained through routine methods (Ben-Shem et al., 2011a; Wells et al., 2020; Kisonaite et al., 2022; Leesch et al., 2023) or to discover a class of dormant ribosomes during analysis of other targeted ribosomal complexes (Anger et al., 2013; Brown et al., 2018; Zhao et al., 2022). To explore further structural details of these dormant states, we elucidated cryo-EM structures of five newly identified Stm1/SERBP1-bound ribosomes, which were 80S•SERBP1•eEF2•eIF5A, 80S•SERBP1•eEF2•E-tRNA, 80S•Stm1•eIF5A, 80S•Stm1•P/E-tRNA, and 80S•Stm1 (Supplementary Figures S1, S2; Supplementary Table S1). These structures presented several new states of ribosomal complexes associated with Stm1/SERBP1 and complemented our understanding of the function of Stm1.

### 3.1 Weak binding of eEF2 in the GAC of the dormant ribosome

**FIGURE 1:**
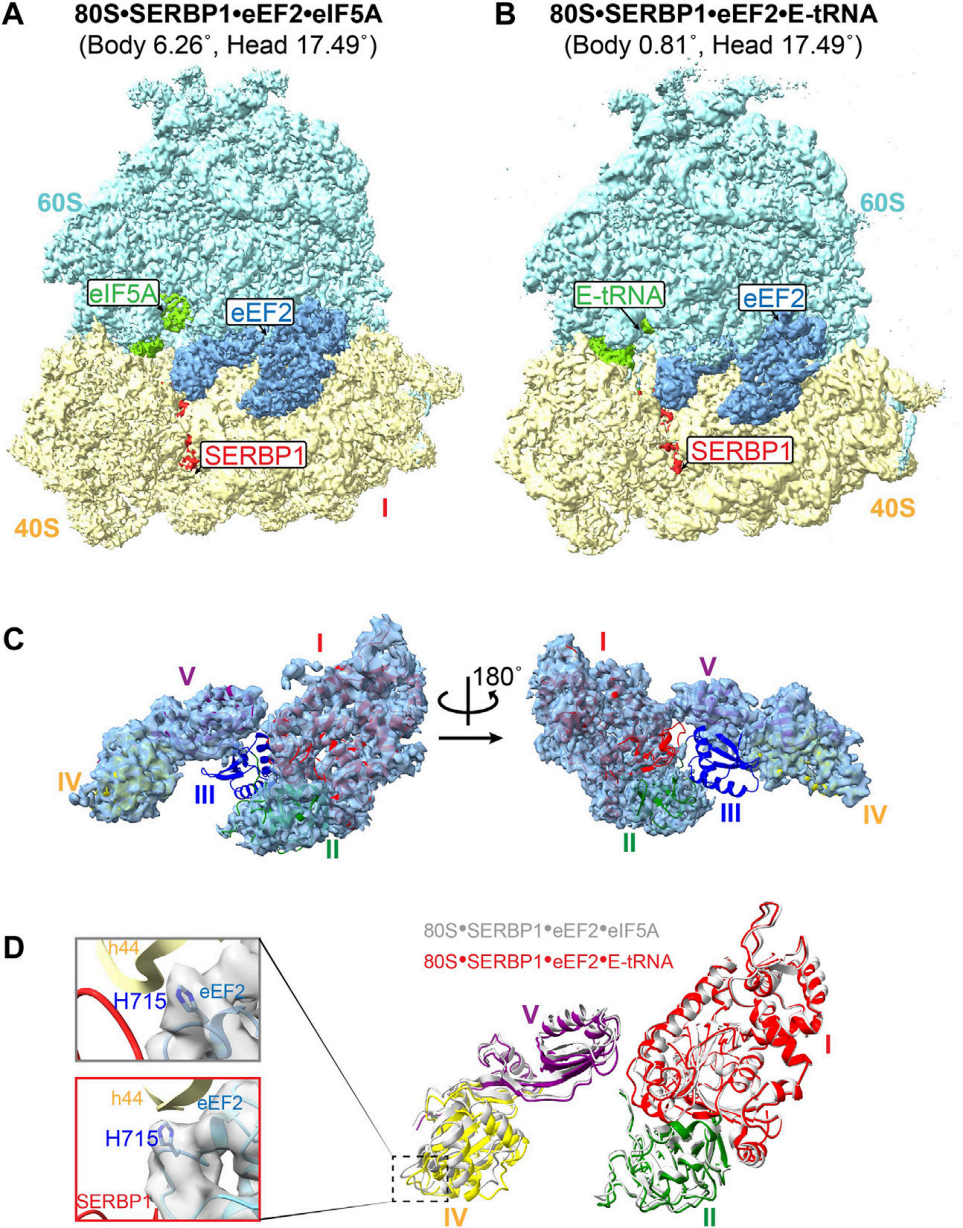
Structure of the human dormant ribosomes. **(A)** and **(B)** the isolated dormant ribosomal particles showed the simultaneous binding of eIF5A **(A)** or E-tRNA **(B)** with eEF2 and SERBP1. **(C)** Domain III of eEF2 is extremely flexible in the complex 80S-SERBP1-eEF2-E-tRNA. **(D)** The movement of domains in associated eEF2 between 80S-SERBP1-eEF2-eIF5A (gray) and 80S-SERBP1-eEF2-E-tRNA (colored). Domain I: red, domain II: green, domain IV: yellow, domain V: purple. Insert on the left: the density maps of H715 in both complexes are shown on the gray surface.

Epitope-based purification of translated products from the cell lysates corresponding cell-free protein synthesis (CFSP) system reflected several dormant ribosomes in combination with the targeted complex. Here, we performed structural analysis on these dormant ribosomes and discovered their predominant binding to SERBP1 (Supplementary Figure S1A). Further classification of these complexes revealed two major conformations, i.e., one bound with SERBP1•eEF2•E-tRNA and the other with SERBP1•eEF2•eIF5A (Figures 1A,B).

The eEF2 protein is comprised of five domains, labeled as I-V, and normally catalyzes the translocation of mRNA and peptidyl-tRNA during translation (Djumagulov et al., 2021). Several dormant ribosome complexes bound eEF2 have already been resolved (Liu and Qian, 2016; Brown et al., 2018). In contrast to previously reported eEF2•80S complexes, both the complexes that we obtained here exhibited significantly high flexibility of domain III of eEF2, with no observed density (Figure 1C; Supplementary Figure S3A). The GTP/GDP binding pocket also lacked density (Figure 1C; Supplementary Figure S3A). Additionally, eEF2 exhibited varying degrees of extension in such a way that domain IV shows a different displacement relative to domain I (Figure 1D). Compared with the resolved structure of 80S•SERBP1•eEF2-E•tRNA (PDB: 6Z6M), distinct movements were also observed (Supplementary Figures S3B, C). These changes could be attributed to the differences in the rotation angles of the 40S body and head (Supplementary Figures S3D, E). When comparing the dormant ribosome-bound eEF2 (Supplementary Figure S4 colored) to whole eEF2 under GTP-form (Supplementary Figure S4 white) and GDP-form (Supplementary Figure S4 gray), we observed that 80S•SERBP1•eEF2•eIF5A had similar conformation as the GDP-form. However, 80S•SERBP1•eEF2•E-tRNA adopted a conformation different from the GTP-form and GDP-form. The increased flexibility in domain III and GTP/GDP-binding pocket and the conformational changes of the eEF2 suggested that eEF2 in our complexes might be in a GTP/GDP-free state.

Previous research demonstrated that the small interface of SERBP1 and eEF2 is unlikely to mediate the anchoring of eEF2 to the ribosome via domain IV (Brown et al., 2018). In another type of dormant ribosomes, the same results have been reported that Habp4 cannot fully stabilize the eEF2 (Leesch et al., 2023). However, the exact mechanism for how the dormant ribosome is able to trap eEF2 is unclear. In addition, a conserved histidine residue of eEF2 (yeast eEF2^His699^) is post-translationally modified to diphthamide, and lack of diphthamide modification decreases the translocation efficiency (Spahn et al., 2004; Taylor et al., 2007; Djumagulov et al., 2021). Structures of human ribosomal complexes with eEF2 and SERBP1 reflected two alternative conformations of diphthamide, which can either establish its interaction with nucleotide A1825 in h44 or SERBP1 within the mRNA path (Anger et al., 2013). However, we did not identify electron density for the diphthamide modification in either of our human dormant ribosomes (Figure 1D). This may indicate that the interaction between diphthamide and SERBP1 is very weak and binding of eEF2 to dormant ribosomes does not require diphthamide. These observations suggested that eEF2 might not play a role in stabilizing the dormant ribosome however, it remains on the ribosome to protect itself from degradation under stress conditions. This notion is consistent with our subsequent observations regarding the binding of eIF5A and tRNA.

### 3.2 Weak binding of eIF5A or tRNA at the E-site of dormant ribosomes

**FIGURE 2:**
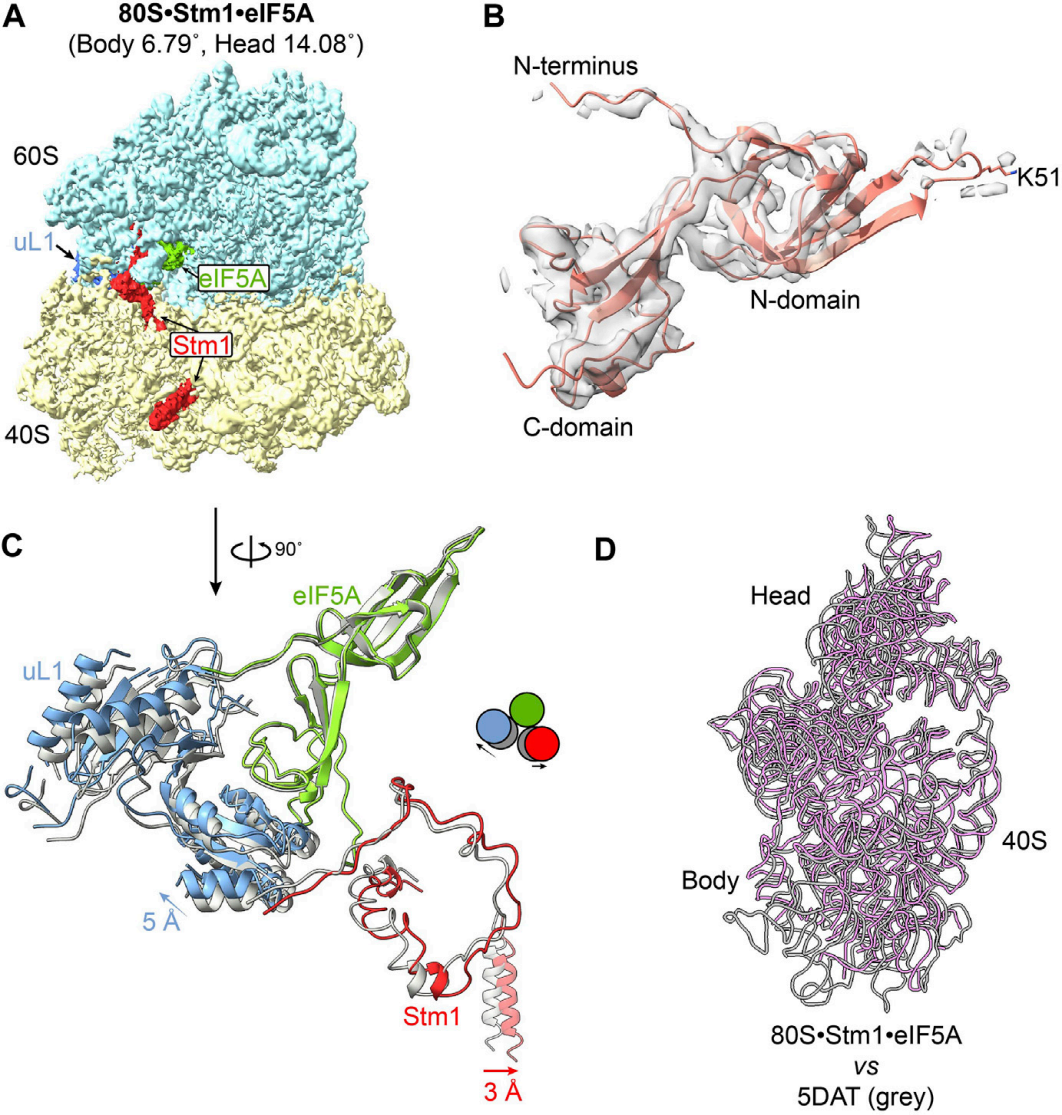
Structure of eIF5A in the 80S-Stm1 complex. **(A)** The overall structure of yeast 80S with Stm1 and eIF5A. Protein eEF2 is absent in this complex. **(B)** The density presented on the gray surface indicates a flexible N-domain in eIF5A in this dormant complex. The residue K51 was shown as unmodified. **(C)** Stm1 (red), eIF5A (green), and uL1 (blue) a exponential compared with the published 80S/eIF5A structure (PDB: 5DAT, shown in gray). **(D)** The movement of 40S body and head; 25S rRNA is aligned in the two complexes. Large subunit and ribosomal proteins in 40S are hidden for clarity. The 18S rRNA for 80S•Stm1•eIF5A in this study is colored in purple, and the previously published one (PDB: 5DAT) is in gray.

eIF5A normally promotes translation elongation and termination, particularly upon ribosome stalling in specific amino acid sequence contexts (Schuller et al., 2017). In addition to the role of eIF5A as a translation factor, it has been reported that dormant ribosomes purified from glucose-starved yeast cells carry Stm1 and eIF5A, although eIF5A dissociates during further treatment (Ben-Shem et al., 2011a; Melnikov et al., 2016). Furthermore, dormant ribosomes purified from egg cells and early embryos have been simultaneously bound by eIF5A and eEF2 (Leesch et al., 2023). Besides 80S•SERBP1•eEF2•eIF5A complex from the 293F cell extract (Figure 1A), we also successfully isolated ribosomal particles containing both Stm1 and eIF5A from exponential phase yeast cells in this study (Figure 2A; Supplementary Figure S1B). In these complexes, eIF5A was bound between the exit (E) and peptidyl (P) site of the ribosome (Figures 1A, 2A), as previously reported for a stalled ribosome (Schmidt et al., 2016). Early studies have suggested that binding of eIF5A to the dormant ribosome is facilitated by cycloheximide (Schmidt et al., 2016). However, recent research conducted by Leesch et al. reveals that four factors, including Habp4, eEF2, Dap1b/Dap, and eIF5A, can simultaneously bind to the same dormant ribosome (Leesch et al., 2023), indicating an alternative possibility underlying the association of eIF5A with the dormant ribosome except for the influence of cycloheximide. In yeast 80S•Stm1•eIF5A structure, the N-terminal domain of eIF5A exhibits poor density, indicating substantial flexibility in the N-terminal region (Figure 2B). In comparison to eIF5A in a translation state resolved from a stalled ribosomal complex (Zeng et al., 2021) (Supplementary Figure S5A), we found that the eIF5A in the Stm1-bound complexes was not hypusinated (Figure 2B). It has been reported that the post-translationally modified hypusine residue stabilized eIF5A’s association with the ribosome (Jao and Chen, 2006). Interestingly, the eIF5A in human 80S•SERBP1•eEF2•eIF5A showed a good N-domain and hypusine (Supplementary Figure S5B). In comparison with the crystal structure (PDB: 5DAT) (Melnikov et al., 2016), our 80S•Stm1•eIF5A complex exhibited a significant shift in uL1, Stm1, and the C-terminal of eIF5A (Figure 2C). This could be attributed to the conformational changes in the 40S subunit (Figure 2D). These observations also highlighted the diverse conformations of yeast dormant ribosomes.

Various tRNAs have been observed to bind at the E-site or in proximity to the dormant ribosome, including E-tRNA, P/E-tRNA, pe/E-tRNA (Kisonaite et al., 2022), and Z-tRNA (Brown et al., 2018). We also obtained a dormant ribosome structure bound with Stm1 (80S•Stm1•P/E-tRNA complex), revealing the presence of P/E-tRNA (Supplementary Figures S1C, S6). The positioning of these tRNAs at the exit site of ribosome suggested that their interaction with the ribosome is highly unstable. This is corroborated by the fact that only a partial density of P/E-tRNA is evident in the 80S•Stm1•P/E-tRNA complex.

### 3.3 The monosomes in cells at the exponential phase primarily comprise dormant ribosomes

**FIGURE 3:**
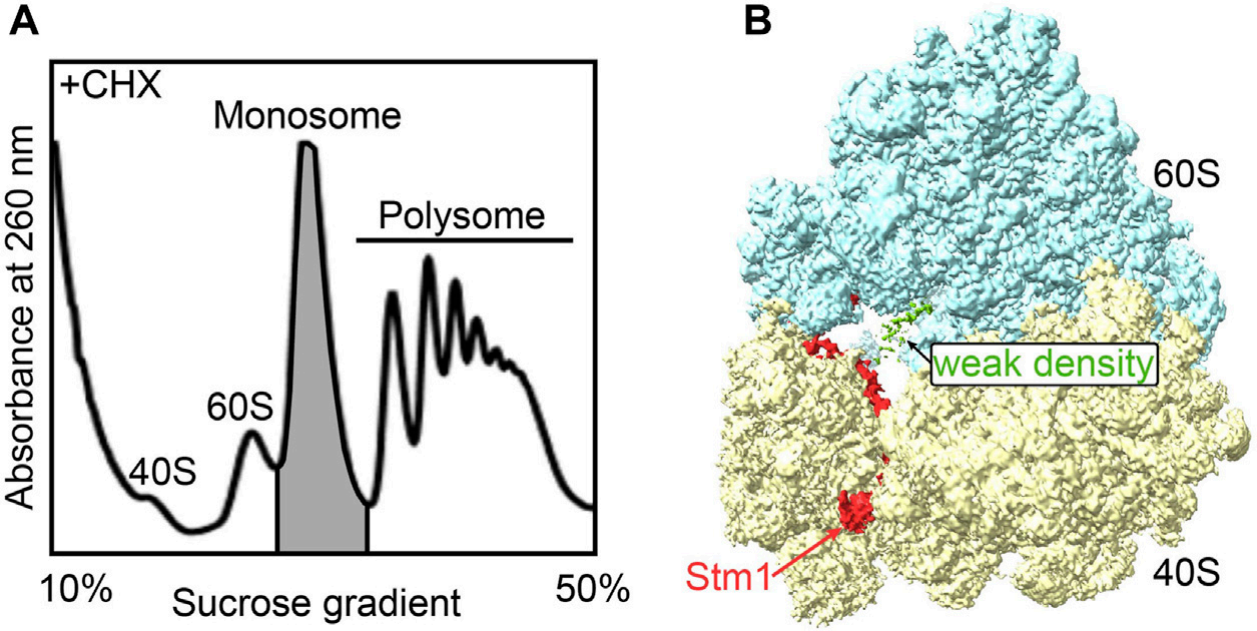
The monosome in active cells is mainly in the Stm1-bound state. **(A)** The isolation of the monosome fractions from the polysome profiling of yeast cells grown in YPED medium with an OD_600_ of 0.6, followed by trapping translation with cycloheximide. **(B)** The structure of the dormant ribosome bound with Stm1 protein.

Many stress conditions cause a global shutdown of translation, allowing cells to economically use limited metabolic resources to produce only the proteins important for adaptation to the changing environment. The formation of dormant ribosomes protects ribosomal subunits from damage and/or degradation (Kraft et al., 2008). What about the ribosomes in the cells growing in the exponential phase? To investigate whether dormant ribosomes exist in cells growing in normal conditions, here, we collected the monosomes from exponential phase yeast cells (BY4742) in the presence of cycloheximide for structural studies (Figure 3A). These structures predominantly captured dormant ribosomes within the monosome peak, although not exclusively (Supplementary Figure S2). Consistent with other dormant ribosomes, clear Stm1 binding to the monosome was observed (Figure 3B). Only a weak electron density was present at the A-site. However, due to its low abundance, neither the local mask in Relion nor CryoDRGN could reliably separate it, which might be probably due to loss of protein or tRNA during purification. Ribo-seq data analysis depicted the existence of some translating ribosome complexes in monosomes (ref). Hence, we speculated that a considerable number of dormant ribosomes co-exist even during the rapid cell growth phase. These dormant ribosomes may serve as a reservoir for a prompt cellular response to external stimuli.

### 3.4 Ribosomes associated with Stm1/SERBP1 exhibit diverse conformations

**FIGURE 4:**
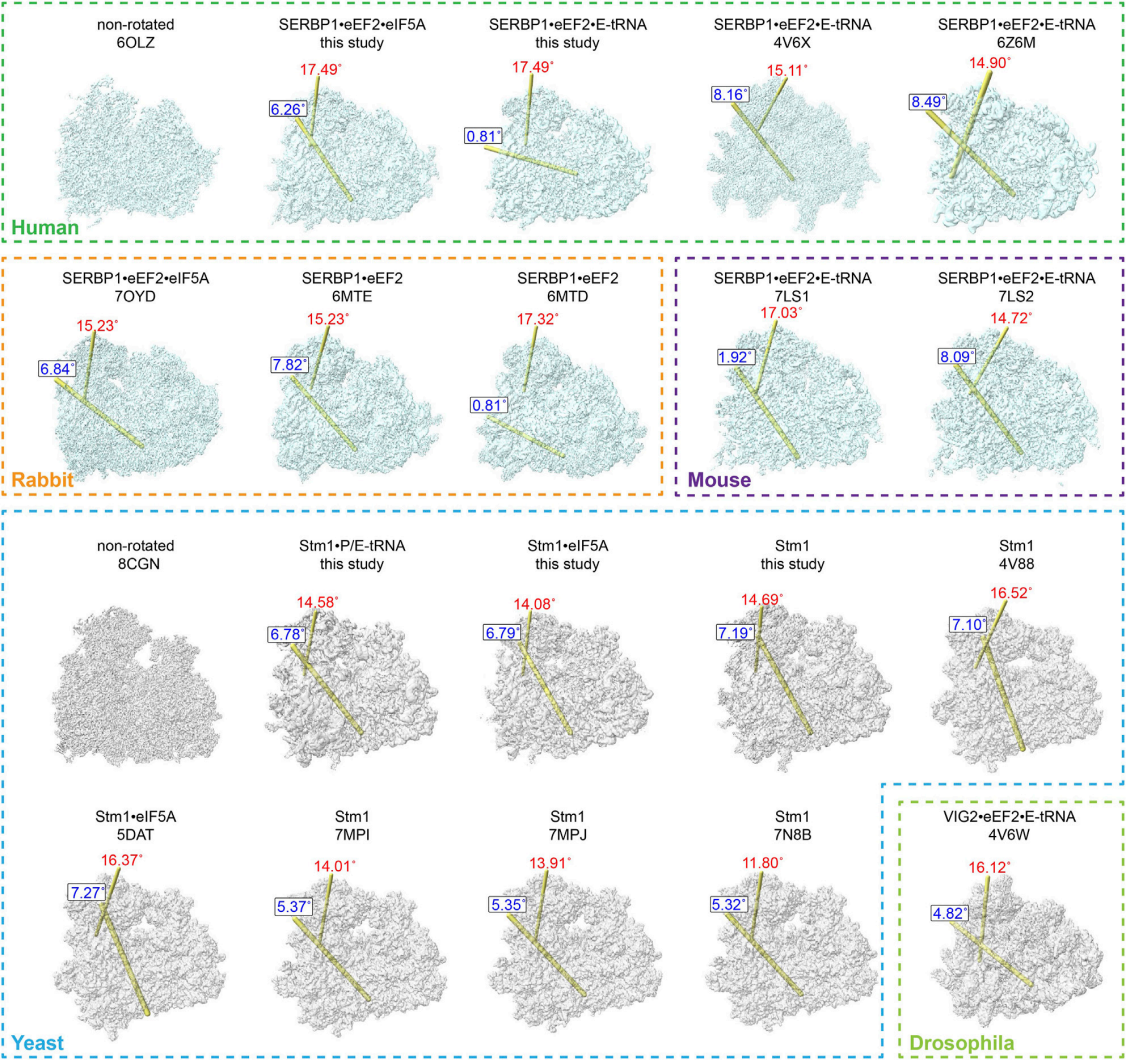
Comparison of dormant ribosome conformations. All the classified and SERBP1/Stm1-bound ribosome conformations were compared with the non-rotated conformation. The ribosomes from humans, rabbits, and mice were aligned with the human non-rotated ribosome (PDB: 6OLZ), while the yeast and *drosophila* ribosomes were aligned with the yeast non-rotated ribosome (PDB: 8CGN). The relationship between any two compared heads is indicated by the location of the rotation axis in yellow and the degree of rotation in red (the swivel). Similarly, the relationship between any two compared bodies is indicated by the location of the rotation axis in yellow and the degree of rotation in blue (ratchet). The numbers indicate the degrees of rotation in a clockwise direction when viewed on each axis.

Various studies reported that Stm1/SERBP1 has been shown to bind to the ribosome in various forms (Ben-Shem et al., 2011b; Anger et al., 2013; Schmidt et al., 2016; Brown et al., 2018; Wells et al., 2020; Smith et al., 2021b; Zhao et al., 2022). It not only binds to the dormant ribosome itself, but also traps eEF2, eIF5A, or E-tRNA. To compare their differences, we summarized the structures of all Stm1/SERBP1-bound 80S ribosomes and calculated/plotted the two rotation axes (Figure 4), previously described as ribosome ratcheting and swiveling (Ling and Ermolenko, 2016). Our results showed that the 40S body rotated from 0° to 10°, with most of the complexes having ratcheting greater than 5°. And the swiveling angle was distributed from 10° to 20° (Supplementary Figure S7). When Stm1 bound to the ribosome alone, we observed a deviation of 11.8°–16.52° in the head region of 40S subunit and a deviation of 5.32°–7.19° in the body region. These results suggested that dormant ribosomes themselves allow slight deviations in the ribosome structure. When the Stm1-bound ribosomes interacted with a single factor, such as P-tRNA or eIF5A (yeast), the deviation angle of 40S subunit decreased as compared to those only have Stm1 bound, with the exception of unrotated state with a swiveled 40S head in 80S•SERBP1•eEF2 (PDB: 6MTD) complex from rabbit. This suggested that binding of P-tRNA and eIF5A did not necessarily fix the conformation of ribosome to a specific state. When the Stm1-bound ribosomes interacted with multiple factors such as eEF2, eIF5A, and tRNA, deviation in the 40S body ranged from 4.82° to 8.49°, while the head ranged from 14.72° to 17.49°. In contrast, two structures including 80S•SERBP1•eEF2•E-tRNA (PDB: 7LS1) and 80S•SERBP1•eEF2•E-tRNA (this study) showed similarities to the rabbit 80S•SERBP1•eEF2 (PDB: 6MTD). Comparative analysis of these states indicated that the interaction with multiple factors did not fix the dormant ribosome into a specific conformation. Also, binding of translation factors did not stabilize the conformation of dormant ribosome. This notion is supported by the instability of interactions between eEF2, eIF5A, tRNA and the dormant ribosome. It is likely that translation factors bind to the dormant ribosome to protect themselves from degradation and facilitate their participation in translation reactions when needed. In other words, one major function of dormant ribosomes is to serve as a pool for storing some of the translation factors.

From these comparisons, we observed distinct patterns within the SERBP1/80S complexes purified from vertebrate cells, which can be categorized into two classes. The first class exhibited no ratcheting but displayed a significant swiveling of approximately 17° (Supplementary Figure S7B). This class included dormant ribosomes from rabbits containing eEF2, mice containing both eEF2 and E-tRNA, as well as our purified human ribosomes containing eEF2 and E-tRNA (Figure 4). In contrast, the other class reflected around 8° of ratcheting and about 15° of swiveling (Supplementary Figure S7B). The latter similarly comprised dormant ribosomes from these three species (Figure 4). Furthermore, it is noteworthy that, even when binding the same proteins, the 40S subunit exhibited distinct rotations in both body and head regions. This observation suggested that the interaction of proteins (eEF2 and eIF5A) and tRNA (E-tRNA) with the ribosome is relatively flexible. In contrast, the angles of ratcheting and swiveling for dormant ribosomes isolated from yeast or non-vertebrate *drosophila* ranged from 4 to 7° and 11° to 16°, respectively (Supplementary Figure S7C). Unlike vertebrates, the distribution of body rotation angles in these dormant ribosomes is more concentrated, while the angles of head rotation are more dispersed. These ribosomes also presented different states of their small subunit, even when the same proteins or tRNA are bound (Figure 4).

In summary, these comparisons suggested: (a). All the dormant ribosomes from the mammalian and *drosophila* cells had eEF2 bound, while none of the yeast ones had eEF2 present. (b). No matter whether the A-site had eEF2 bound or was empty, the E-site could be empty or had eIF5A or tRNA bound. (c). No matter in which species, the dormant ribosomes had different ratcheting and swiveling angles for the small subunits, even though they had the same contents in the A- and E-site. (d). The dormant ribosomes from different species could have the same ratcheting and swiveling angles for the small subunits or have the same conformation of eEF2. (e). Even though the ratcheting and swiveling angles for the small subunits were the same, the conformation of bound factors could be different. These results collectively demonstrate the conformational diversity of dormant ribosomes and the associated proteins/tRNAs, suggesting a similar function of Stm1/SERBP1-bound dormant ribosomes in different eukaryotic cells, which is protecting ribosome itself and also some translation factors against degradation.

### 3.5 Depletion of Stm1 affects the expression of genes in metabolic and DNA integration

Combined with the previous reports (Anger et al., 2013; Brown et al., 2018; Kisonaite et al., 2022; Leesch et al., 2023), our current work reflected that Stm1 binds to dormant ribosomes with different factors. These structures highlighted that the C-terminal of Stm1 exhibits a minimal function in ribosomes. However, it has been observed that six deletions in Stm1 (∆67–74, ∆102–106, ∆169–172, ∆174–184, ∆188–194, and ∆240–244) have failed to rescue the temperature-sensitive phenotype of ∆*pat1* and failed to inhibit the growth of a ∆*dhh1* strain, indicating that the entire protein is required for optimal function (Balagopal and Parker, 2011). To investigate the functions of Stm1 beyond its involvement in dormant ribosomes, we retrieved Ribo-seq data from Egorov et al. (GSE185458), which has not been analyzed yet, and performed a deep analysis (Supplementary Figure S8; Supplementary Table S2) (Egorov et al., 2021). For that purpose, comparisons of transcription and translation levels between ∆*stm1* and wild-type (WT) yeast cells were performed.

**FIGURE 5:**
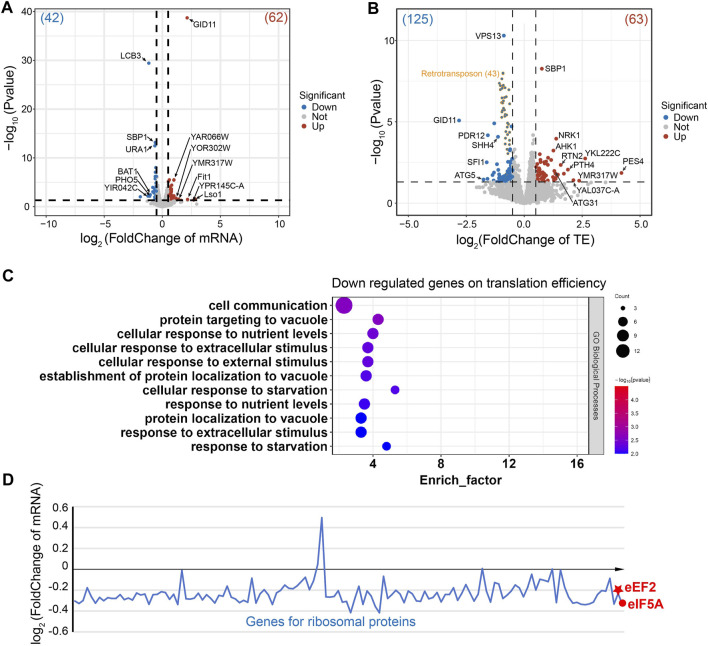
Gene regulation of the ∆stm1 cells. **(A)** and **(B)** volcano plots showing the fold changes in mRNA on the transcription level **(A)** or the translation efficiency (TE). **(B)** The numbers of regulated genes are shown in parentheses. The 43 retrotransposon TYA Gag and TYB Pol genes are labeled in orange. **(C)** The GO pathway enrichment analysis of the downregulated genes on the translation efficiency indicated enrichments in the nutrient or stimulus processes. **(D)** Distribution of the mRNA fold change of ribosomal proteins and eEF2 and eIF5A.

In the ∆*stm1* strain, only four genes (LCB3, Pho5, BAT1, and an uncharacterized protein) at the transcription level were downregulated more than 2-folds (Figure 5A). LCB3 is a long-chain base-1-phosphate phosphatase that regulates ceramide and long-chain base phosphates levels, which are involved in the incorporation of exogenous long-chain bases into sphingolipids (Zhang et al., 2001). PHO5 is a repressible acid phosphatase and mediates extracellular nucleotide-derived phosphate hydrolysis (Kennedy et al., 2005). BAT1 is a mitochondrial branched-chain amino acid (BCAA) aminotransferase (Koonthongkaew et al., 2020). In contrast, seven genes were upregulated more than 2-folds (Figure 5A), involving Lso1 and Fit1, two iron-homeostasis mediated proteins (Yun et al., 2000; An et al., 2015), and GID11, substrate receptor of glucose-induced degradation-deficient (GID) complex (Fechtner and Pfirrmann, 2021), while the other genes were uncharacterized. Despite differences in their specific functions, these genes are collectively involved in cellular metabolism and regulatory processes, suggesting the importance of Stm1 in mediating diverse regulatory processes such as lipid metabolism, phosphate hydrolysis, branched-chain amino acid metabolism, and iron ion homeostasis. Interestingly, Lso2 (Lso1 paralog) could bind to dormant ribosomes (Wang et al., 2018; Wells et al., 2020). Given the high level of sequence similarity between Lso1 and its paralog Lso2 (Supplementary Figure S9A), Lso1 showed a similar structure to dormant ribosome-bound Lso2 based on the alphaFold results (AFDB accession code: AF-Q3E827-F1), reflecting that Lso1 might have a similar function to Lso2 in dormant ribosomes (Supplementary Figure S9B). The upregulated level of Lso1 in ∆*stm1* cells may suggest an interplay between Stm1 and Lso1. To gain more insights into this regulation, we set the cutoff value of log_2_(FoldChange) to ±0.5, and observed that 42 genes were downregulated (>1.4 fold), and GO analysis showed that they were enriched in the metabolic pathway and biosynthesis of co-factors, or some ion transport processes (Supplementary Figure S10). In addition, 62 genes were upregulated (<0.7 fold), which were enriched in metal ion transport pathways as well (Supplementary Figure S11).

Initially, Stm1 was reported to be able to associate with polysomes (Van Dyke et al., 2006), but recent studies have shown that its binding to polysomes may be non-specific or unstable, as it can be abolished by 150 mM KCl or higher, while its binding to 80S ribosome can be disrupted by 300 mM KCl (Shetty et al., 2023a). Nevertheless, the Ribo-seq data showed that the translation efficiencies of 20 genes are downregulated and 23 genes are upregulated more than 2-folds (Figure 5B). These regulations may be directly or indirectly affected by the translation initiation/elongation via Stm1 or some Stm1-targeting genes, respectively. Three of the downregulated genes are directly related to translation, including PES4, a putative RNA-binding protein related to translational timing and localization; PTH4, a stalled-ribosome rescue factor; and RSA1, which is involved in the assembly of 60S ribosomal subunits. An unknown function protein, YKL222C, may also interact with ribosomes based on co-purification experiments, while RTN2 and ATG31 were involved in tubular ER morphology and autophagy, respectively (Figure 5B). Among the upregulated genes, GID11 is particularly noteworthy, as this protein exhibits increased expression at the mRNA level and significant downregulation at the translation level (Figure 5B). This implies a close association between the GID1 concentration and ribosome preservation factor Stm1. Other genes showing an increase in translation efficiency included autophagy-related ATG5, spindle-pole-related SFI1, and several transcription factors (Figure 5B). Based on log_2_(FoldChange) as ± 0.5, 63 downregulated and 125 upregulated genes were filtered. GO analysis for the downregulated genes reflected enrichment in cell processes such as metabolism, translation, and gene expression (Supplementary Figure S12). In contrast, 43 of the upregulated genes are retrotransposon TYA Gag and TYB Pol genes. The GO analysis of the rest of 82 genes generated an enrichment in cell communication and response to nutrient levels or external stimuli (Figure 5C). Together, these results suggested that Stm1 can directly regulate the expression of nutrition-related genes, enabling cells to cope with the growth in nutrient-deprived environments, rather than solely serving the conventional role of protecting idle ribosomes from degradation. However, more experiments are required to determine the regulation mechanism of Stm1.

To check the changes of eEF2, eIF5A, and ribosomal proteins, the fold changes of mRNA level were plotted for these genes (Figure 5D). We observed that most of the mRNAs encoding ribosomal protein decreased the level to about 85% (log_2_(FoldChange) is about −0.2) in ∆*stm1* cells compared to WT cells. For eEF2 and eIF5A, the log_2_(FoldChange) is −0.23 and −0.33, respectively. The decrease of mRNA level for these genes suggested that they are submitted to degradation when Stm1 is absent. We then hypothesized that under stress conditions, Stm1/SERBP1 first traps the 60S and 40S as a dormant ribosome, which protects the ribosome against degradation. At the same time, with the additional interactions between Stm1/SERBP1 and eEF2, eIF5A, and tRNA, these factor binds weakly to the dormant ribosome, resulting in protection of themselves against degradation. The weak binding ensures the rapid release from dormant ribosomes when they are needed for translation. While in ∆*stm1* cells, Stm1-mediated dormant ribosomes could not be formed, and they will be marked for degradation. Same for the eEF2 and eIF5A. When Stm1 is absent, they can not bind to the empty ribosome and will be marked for degradation.

## 4 Discussion

Deciphering the molecular mechanisms of the ribosome has been a central theme in molecular biology for decades. Recent research also focuses on how dormant ribosomes regulate the translation status under stress conditions or during the embryonic period. Here, we identified five new dormant ribosomes from yeast and human cells. The structural analysis suggested that Stm1 may protect eEF2, eIF5A, and tRNA from degradation through dormant ribosomes when not in use.

The rotation of ribosomal small subunit often represents different translation states such as classical pre-translocation, translocation and post-translocation states (Carbone et al., 2021). Although the Stm1-bound 80S ribosomes do not actively participate in translation, the distribution of ratcheting and swiveling angles suggests that the 40S subunit of these ribosomes also undergoes varying degrees of rotation. Apart from the two non-rotated and slightly rotated dormant ribosomes observed in *C. thermophilum* (Kisonaite et al., 2022), which may be related to the higher stability of ribosome in thermophiles, dormant ribosomes bound by Stm1/SERBP1 are predominantly in a rotated state (Ben-Shem et al., 2011a; Anger et al., 2013; Brown et al., 2018; Wells et al., 2020; Zhao et al., 2022; Leesch et al., 2023). Whereas the Lso2/CCDC124-bound 80S, the other distinguishable population of dormant 80S that exists in eukaryotes, were found in the non-rotated state, similar to the post-translocational state with tRNAs at P and E sites and an empty A site (Wells et al., 2020). Our analysis revealed that the body-ratcheting angles of yeast dormant ribosomes are relatively concentrated, while the head-swiveling angles have a larger distribution. Dormant ribosomes in humans, rabbits and mice can be mainly categorized into two classes. For instance, one without body rotation but significant head swiveling and the other with body-ratcheting angles similar to yeast but more concentrated at around 15° of head swiveling. It is noteworthy that ribosomes with different rotational angles can bind to the same proteins or tRNAs. These diverse states indicate that the dormant ribosomes bound by Stm1/SERBP1 in eukaryotes do not stably bind translation factors like eIF5A. In fact, binding of Stm1 itself to the ribosome also exists in different states. The dynamics of Stm1 in terms of ribosomal 40S head swiveling have been visualized (Kisonaite et al., 2022). Interestingly, the dormant ribosomes obtained showed similar ratcheting and swiveling upon harvesting the yeast cells at both OD_600_, i.e., 0.6 (this study) and 1.5 or12-16 (Ben-Shem et al., 2011a; Zhao et al., 2022), suggesting that the rotations of 40S head and body in dormant ribosomes might not be affected by the cell growth conditions. However, different ribosome purification methods can cause differences in the ribosomal complexes. For instance, both 80S•Stm1•P/E-tRNA and 80S•Stm1 complexes were purified from yeast cells grown in the exponential phase. Cell harvesting was performed at 4°C for 10 min without adding CHX for 80S•Stm1•P/E-tRNA, while at 30°C for 1 min with CHX present for 80S•Stm1. In addition, disruption of cells was slow for 80S•Stm1•P/E-tRNA (about 5 min) and very quick for 80S•Stm1 (about 1 min). Comparative analysis of these two methods reflected that the slow procedure generated stress for the cells, so tRNA was bound to the dormant ribosome, however, the quick procedure generated dormant ribosomes without binding any factors except Stm1. These results suggested that under distinct stress conditions, dormant ribosomes could be different.

To date, eEF2 has been found to bind to the GAC of dormant ribosomes associated with Stm1/SERBP1, while eIF5A and tRNA can bind to its E-site or its close proximity (Anger et al., 2013; Brown et al., 2018; Wells et al., 2020; Smith et al., 2021a; Kisonaite et al., 2022; Leesch et al., 2023). Binding of these proteins and tRNA to dormant ribosomes can occur either independently or simultaneously. As illustrated in Figure 4, the binding of these factors did not show a strong correlation with the state of the ribosomal small subunit. In yeast, Stm1 could stabilize eEF2 in both GTP- and GDP-states regardless of the diphthamide modification (Hayashi et al., 2018), but there have been no reports of yeast Stm1/80S binding to eEF2. In our study, domain III of eEF2 and the nucleotide-binding site exhibited significant flexibility, and diphthamide could not be tracked. Meanwhile, eIF5A and tRNA showed poor density, indicating relatively weak binding to the ribosome. Therefore, a straightforward conclusion is that the binding of tRNA, eIF5A, and eEF2 to dormant ribosomes is merely to avoid degradation, enabling rapid deployment by the translation machinery. In the monosome peak of cells grown in the exponential phase that we collected with a “quick” method, only Stm1 was bound, suggesting that during the rapid growth phase, these proteins or tRNA are fully utilized, while there is some redundancy in ribosomes. However, why other translation factors do not appear on the ribosome remains a question to be answered.

Shetty et al., 2023b demonstrated that under nutrient-sufficient conditions, TORC1 phosphorylates Stm1/SERBP1 at serines 41 and 55 to prevent it from forming a complex with the 80S ribosome. Upon TORC1 inhibition, dephosphorylated Stm1/SERBP1 forms non-translating, dormant 80S ribosomes that are protected from proteasome-mediated degradation (Shetty et al., 2023a). While the stabilization of dormant ribosomes by Stm1 has been repeatedly demonstrated, the precise regulatory mechanisms through which Stm1 dissociates from the ribosome, thereby allowing dormant ribosomes to re-enter the translation cycle, remains enigmatic. Additionally, some studies have indicated that Stm1 can reduce binding of eEF3 to ribosome and inhibit translation, which aligns well with the mechanisms involved in dormant ribosomes. The translation repression mediated by Stm1 could be antagonized by eEF3 (Hayashi et al., 2018). However, the means by which eEF3 can alleviate Stm1’s inhibitory effect on translation remains unexplained. eEF3 is a fungal-specific elongation factor that binds to the CP of the large subunit and head of the small subunit while the ribosome is in a non-rotated state. This binding facilitates opening of the L1 stalk, releasing eEF3 and promoting translation (Ranjan et al., 2021). Structurally, there is no spatial conflict between the binding sites of eEF3 and Stm1. The question arises: does the reuse of dormant ribosomes have a connection with the ability of eEF3 to alleviate the inhibitory action of Stm1 and facilitate the dissociation of Stm1 from the ribosome? Further experiments are required to address this issue comprehensively.

In addition to its ability to bind the dormant ribosomes, Stm1/SERBP1 exhibits various other functions. SERBP1 has been reported to preferentially bind to GC-rich motifs (Kosti et al., 2020), and subsequent studies have revealed that these motifs could include G-quadruplexes (Su et al., 2021). Additionally, SERBP1 interacts with arginine-methylated and stress-granule-associated proteins (Youn et al., 2018). Similarly, Yeast Stm1 has also been demonstrated to bind to DNA and RNA G-quadruplexes *in vitro* (Yan et al., 2021). Dhh1 and Pat1 proteins, which promote decapping *in vivo*, function in part to directly repress translation initiation (Coller and Parker, 2005; Nissan et al., 2010). Observations have indicated that specific deletions in Stm1 fail to rescue the temperature-sensitive phenotype of the ∆*pat1* strain and are ineffective in inhibiting the growth of ∆*dhh1* strain (Balagopal and Parker, 2011). These functions are unrelated to Stm1’s ability to bind dormant ribosomes. In addition, we analyzed the Ribo-seq data from ∆*stm1* (Egorov et al., 2021), and confirmed that Stm1 regulated the gene expression levels of multiple cellular processes, including those related to nutrient levels, external stimuli, and metabolism. These processes collectively demonstrated Stm1’s protective role in cells under stressful conditions. *In vitro* and *in vivo* studies have shown that the expression level of SERBP1 impacts various cancer-related phenotypes, including stemness, neuronal differentiation, and tumor growth, while the knockdown of SERBP1 affects the expression of genes linked to neurogenesis and synaptogenesis (Kosti et al., 2020). However, some of the differences in the RNA-seq/Ribo-seq data for the ∆*stm1* cells may not reflect Stm1-regulated events, but, rather, the compensatory mechanisms that emerge in the ∆*stm1* cells. More experiments are needed to determine the regulatory mechanism of Stm1.

## Data Availability

The datasets presented in this study can be found in online repositories. The names of the repository/repositories and accession number(s) can be found in the article/Supplementary Material. Electron microscopy maps have been deposited in the Electron Microscopy Data Bank under accession codes EMD-37991 and EMD-37992 for human 80S•SERBP1•eEF2•eIF5A and 80S•SERBP1•eEF2•E-tRNA, respectively, and EMD-37993 for yeast 80S•Stm1, EMD-37994 for yeast 80S•Stm1•E-tRNA, and EMD-37995 for yeast 80S•Stm1•eIF5A. Coordinates have been deposited in RCSB under accession codes 8Y0W and 8Y0X for human 80S•SERBP1•eEF2•eIF5A and 80S•SERBP1•eEF2•E-tRNA, respectively, and 8Y0U for yeast 80S•Stm1•eIF5A.
