# Supplementary material for: Implication of Stm1 in the protection of eIF5A, eEF2 and tRNA through dormant ribosomes
Source: Front Mol Biosci. 2024 Apr 18;11:1395220. doi: 10.3389/fmolb.2024.1395220 (PMC11063288; doi:10.3389/fmolb.2024.1395220)
Supplement: Supplementary file 1 [file DataSheet1.zip › Figure S1_new.pdf]

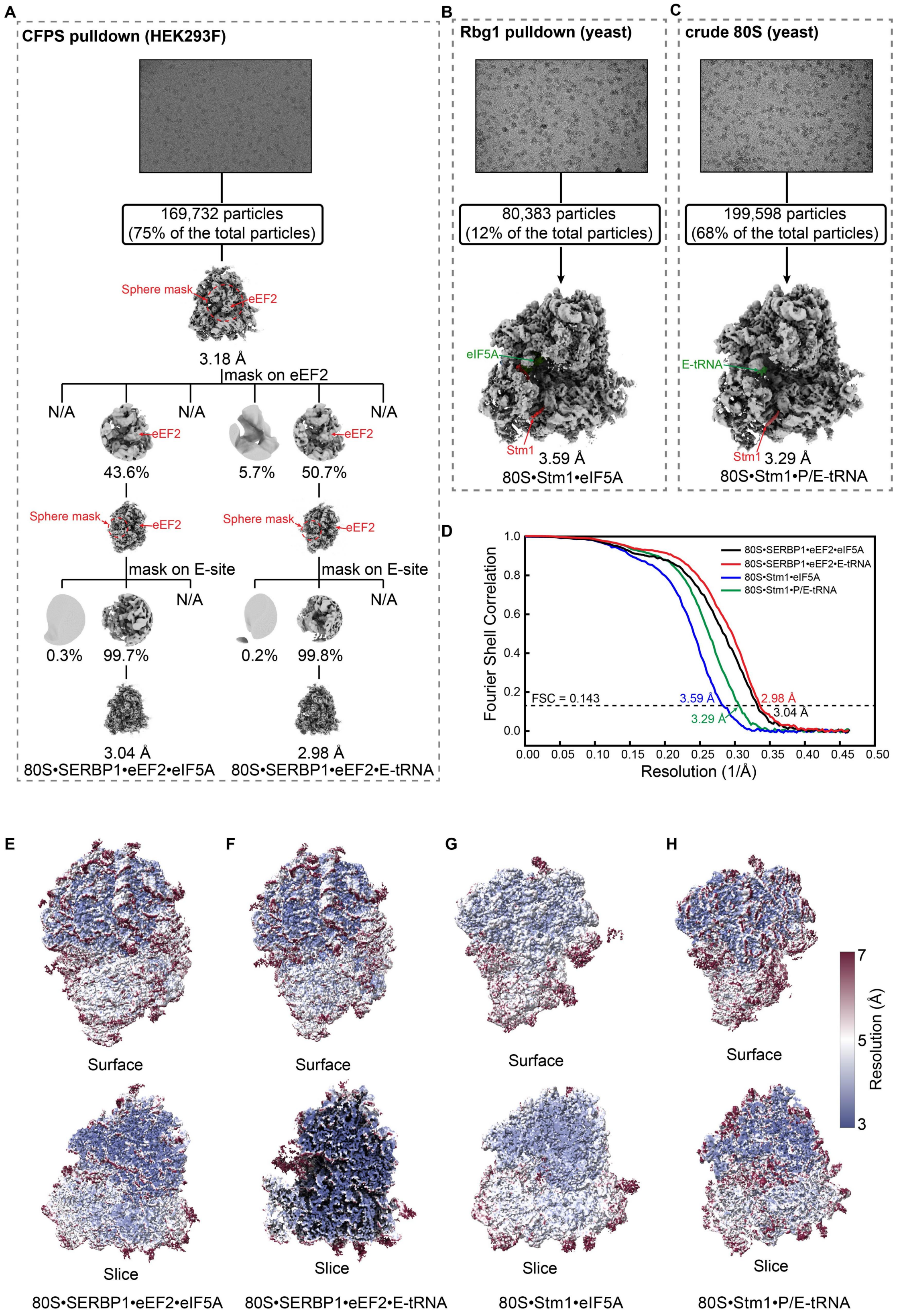

Figure S1. Structure determination of the dormant ribosomes. A. Particle classification and structure refinement procedures used for isolating the two eEF2 containing dormant ribosomes from HEK293F cells. **B** and **C**. Data processing for the eIF5A or E-tRNA bound dormant ribosomes from yeast, respectively. **D**. Gold-standard FSC curves for the electron microscopy maps. Resolutions are

demarcated using the FSC=0.143 criterion. **E-H**. Local-resolution-filtered maps of the dormant ribosomes. Maps are colored according local resolution.
