# Supplementary material for: Implication of Stm1 in the protection of eIF5A, eEF2 and tRNA through dormant ribosomes
Source: Front Mol Biosci. 2024 Apr 18;11:1395220. doi: 10.3389/fmolb.2024.1395220 (PMC11063288; doi:10.3389/fmolb.2024.1395220)
Supplement: Supplementary file 1 [file DataSheet1.zip › Figure S2_new.pdf]

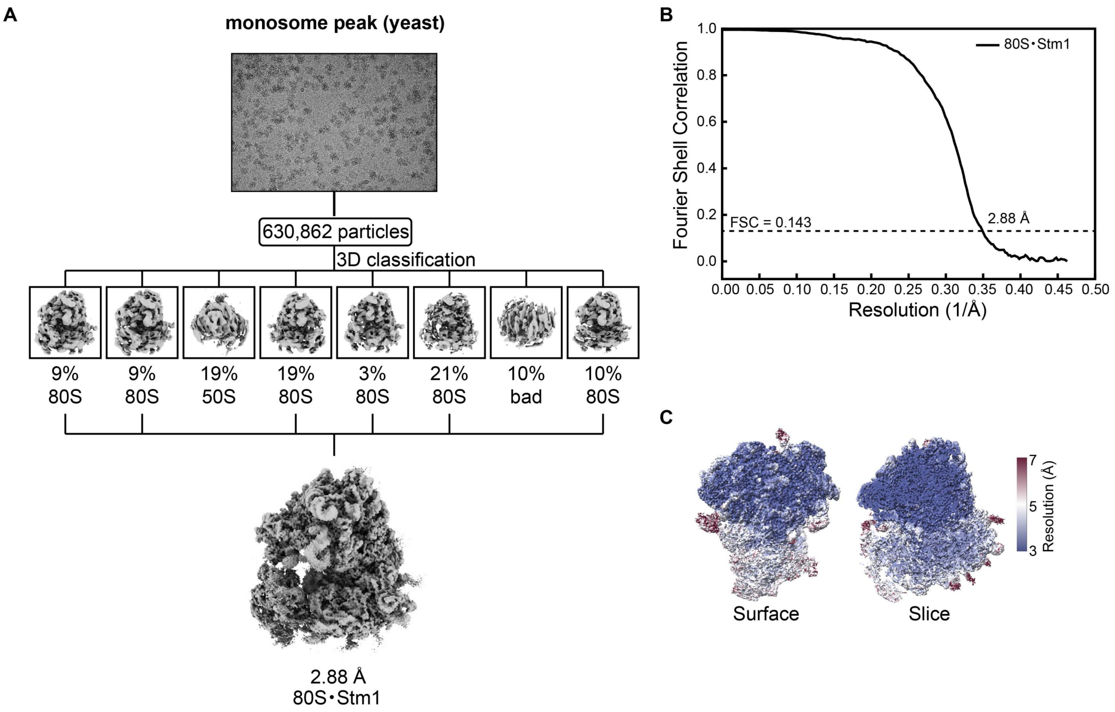

Figure S2. Determination of the ribosomal structure in the monosome peak of yeast polysome profiling. A. Particle classification and structure refinement procedures. **B**. Gold-standard FSC curves for the electron microscopy maps. Resolusions are demarcated using the FSC=0.143 criterion. **C**. Local-resolution-filtered maps, colored according local resolution.
