# Supplementary material for: Implication of Stm1 in the protection of eIF5A, eEF2 and tRNA through dormant ribosomes
Source: Front Mol Biosci. 2024 Apr 18;11:1395220. doi: 10.3389/fmolb.2024.1395220 (PMC11063288; doi:10.3389/fmolb.2024.1395220)
Supplement: Supplementary file 1 [file DataSheet1.zip › Figure S3.pdf]

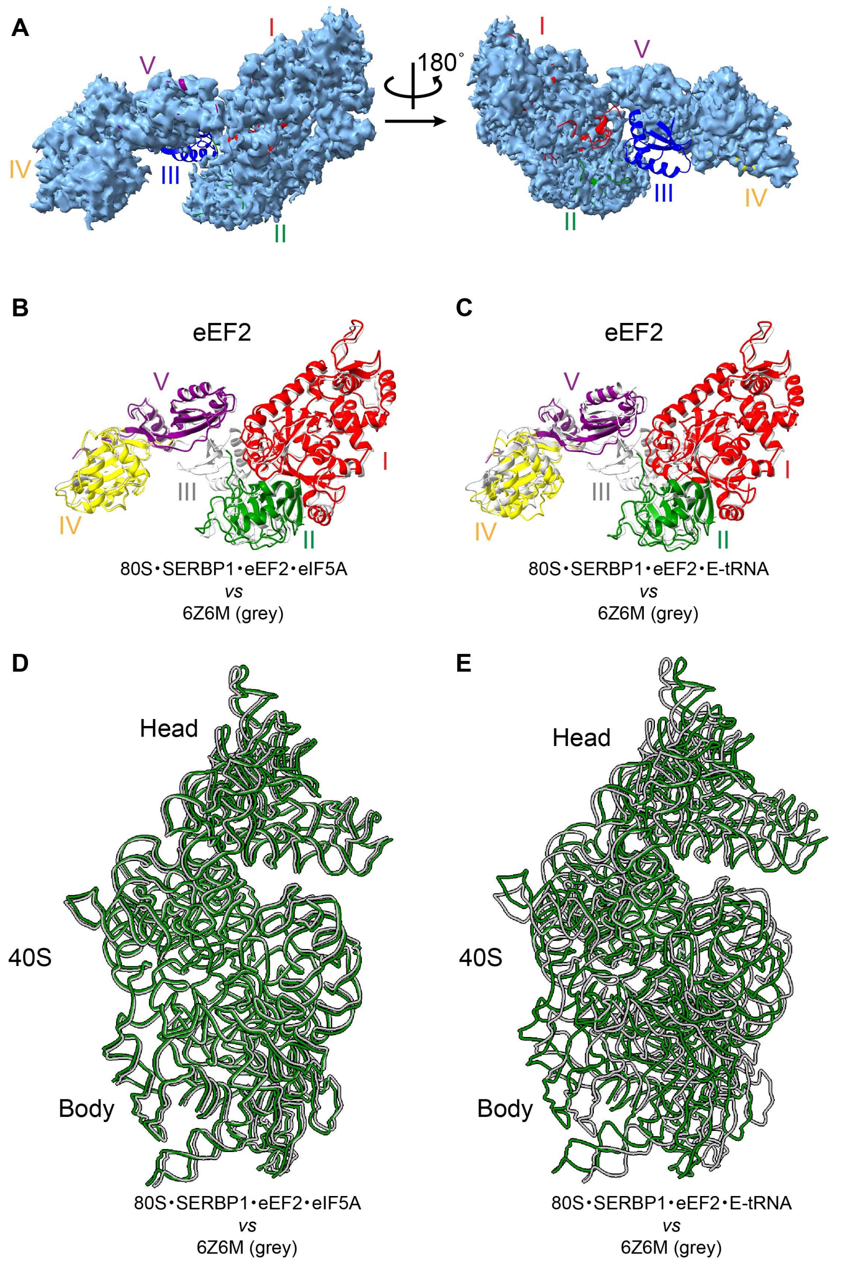

Figure S3. The two human dormant ribosomal complexes showed different conformation. A. The density map shown in blue surface indicated a flexible domain III of eEF2 in complex 80S•SERBP1•eEF2•eIF5A. **B** and **C**. Comparision of eEF2 in the dormant ribosomes obtained in this study to the one in published dormant ribosomal complex (PDB: 6Z6M). **D** and **E**. Comparision of 40S with previously published dormant ribosome (PDB: 6Z6M). All the comparisions were based on the alignment of 28S rRNA. Ribosomal proteins, rRNA and other components were removed for clarity.
