# Supplementary material for: Implication of Stm1 in the protection of eIF5A, eEF2 and tRNA through dormant ribosomes
Source: Front Mol Biosci. 2024 Apr 18;11:1395220. doi: 10.3389/fmolb.2024.1395220 (PMC11063288; doi:10.3389/fmolb.2024.1395220)
Supplement: Supplementary file 1 [file DataSheet1.zip › Figure S4_new.pdf]

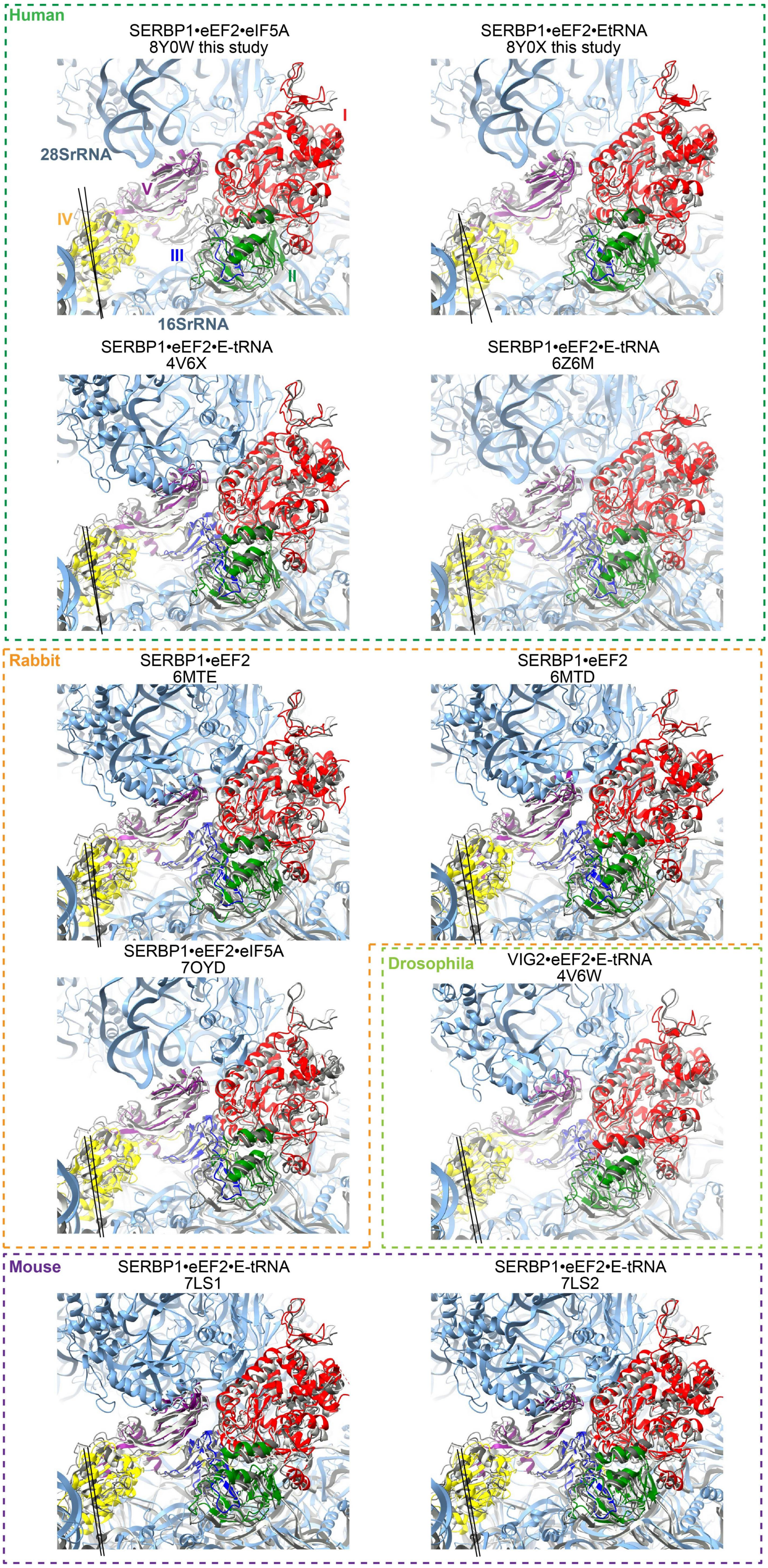

Human
SERBP1•eEF2•eIF5A
8Y0W this study
SERBP1•eEF2•EtRNA
8Y0X this study
28SrRNA
I
V
IV
III
II
16SrRNA
SERBP1•eEF2•E-tRNA
4V6X
SERBP1•eEF2•E-tRNA
6Z6M
Rabbit
SERBP1•eEF2
6MTE
SERBP1•eEF2
6MTD
SERBP1•eEF2•eIF5A
7OYD
Drosophila
VIG2•eEF2•E-tRNA
4V6W
Mouse
SERBP1•eEF2•E-tRNA
7LS1
SERBP1•eEF2•E-tRNA
7LS2

Figure S4. The comparison of eEF2 in different dormant ribosomes (colored) with GTP-form (white) and GDP form eEF2 (gray).
