# Supplementary material for: Implication of Stm1 in the protection of eIF5A, eEF2 and tRNA through dormant ribosomes
Source: Front Mol Biosci. 2024 Apr 18;11:1395220. doi: 10.3389/fmolb.2024.1395220 (PMC11063288; doi:10.3389/fmolb.2024.1395220)
Supplement: Supplementary file 1 [file DataSheet1.zip › Figure S5_new.pdf]

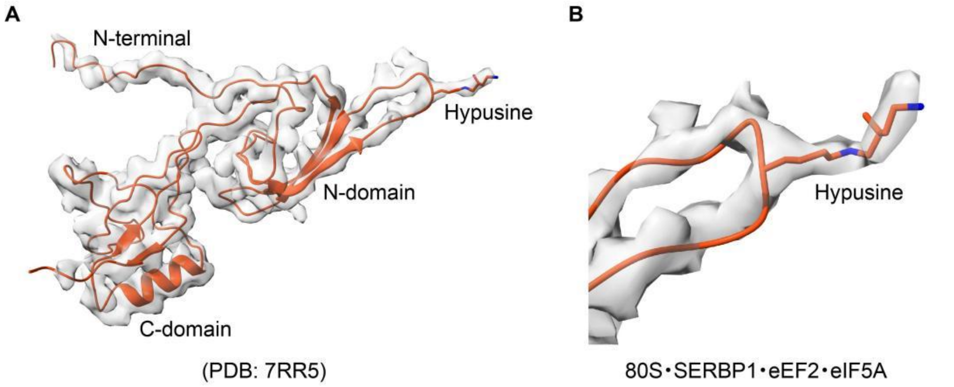

Figure S5. Hypusine modification of eIF5A in the ribosomes. A. The functional eIF5A on the stalled ribosomes mediated by Rbg1/Tma46 (PDB: 7RR5) is hypusine modified. **B**. The eIF5A on the human dormant ribosomal complex 80S•SERBP1•eEF2•eIF5A is also hypusine modified. Density map were shown as grey surface and eIF5A were presented as cartoon in red.
