# Supplementary material for: Implication of Stm1 in the protection of eIF5A, eEF2 and tRNA through dormant ribosomes
Source: Front Mol Biosci. 2024 Apr 18;11:1395220. doi: 10.3389/fmolb.2024.1395220 (PMC11063288; doi:10.3389/fmolb.2024.1395220)
Supplement: Supplementary file 1 [file DataSheet1.zip › Figure S6_new.pdf]

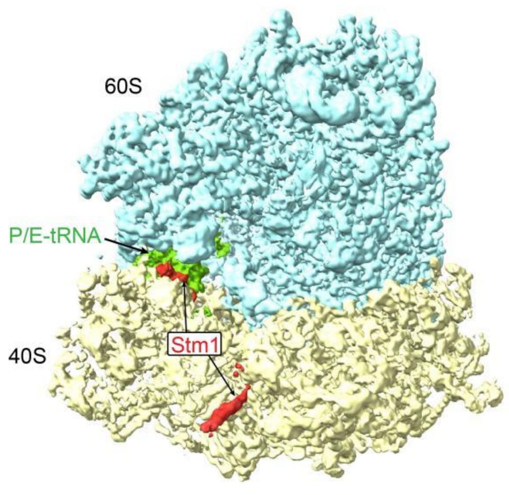

Figure S6. Overall reconstruction of the 80S•Stm1•P/E-tRNA complex. Stm1 and P/E-tRNA were represented in red and green, respectively.
