# Supplementary material for: Implication of Stm1 in the protection of eIF5A, eEF2 and tRNA through dormant ribosomes
Source: Front Mol Biosci. 2024 Apr 18;11:1395220. doi: 10.3389/fmolb.2024.1395220 (PMC11063288; doi:10.3389/fmolb.2024.1395220)
Supplement: Supplementary file 1 [file DataSheet1.zip › Figure S7_new.pdf]

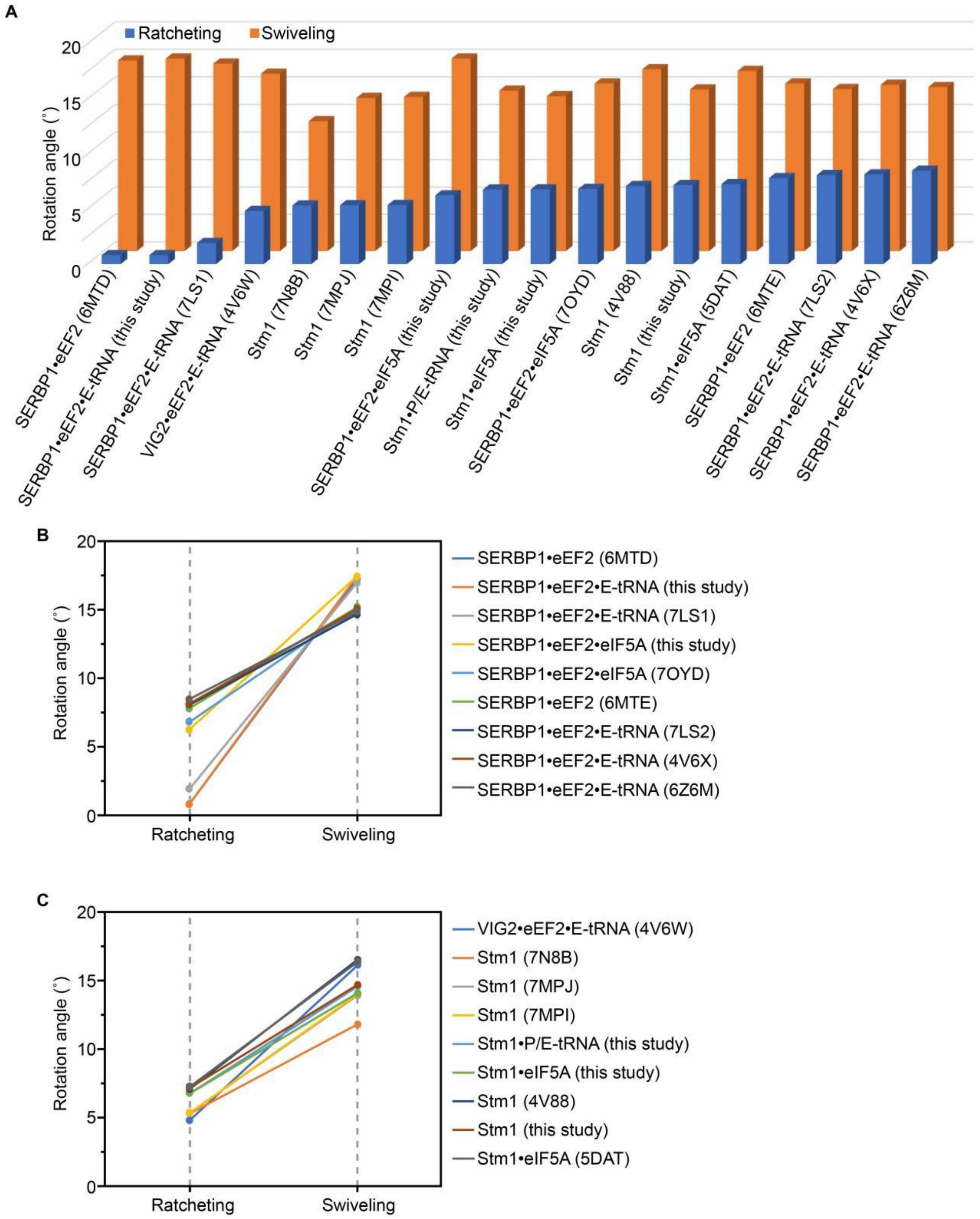

Figure S7. Distribution of the 40S ratcheting and swiveling angles. A. All angles were shown in bar plot and sorted on ratcheting angle. **B**. Clustering of the 40S rotation for dormant ribosomes in human, rabbit, and mouse. **C**. Clustering of the 40S rotation for dormant ribosomes in yeast and drosophila.
