# Supplementary material for: Implication of Stm1 in the protection of eIF5A, eEF2 and tRNA through dormant ribosomes
Source: Front Mol Biosci. 2024 Apr 18;11:1395220. doi: 10.3389/fmolb.2024.1395220 (PMC11063288; doi:10.3389/fmolb.2024.1395220)
Supplement: Supplementary file 1 [file DataSheet1.zip › Figure S8_new.pdf]

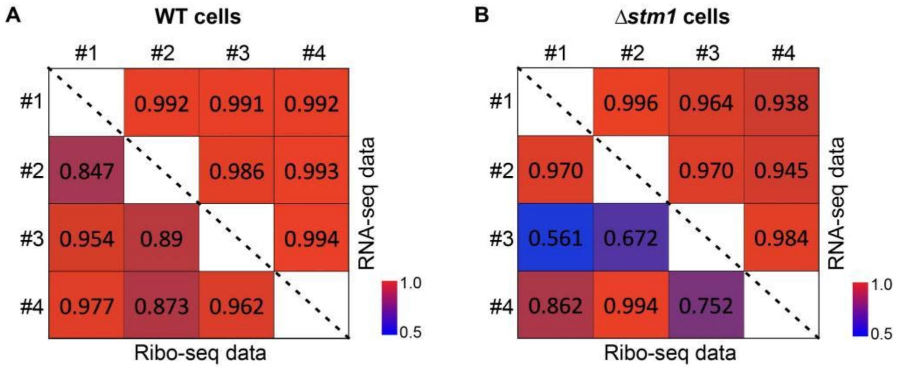

Figure S8. The correlation of high-throughput data in repeated experiments. The correlation factor of TPM (transcripts per kilobase million) for each datasets was calculated by scatter plot and fitted linearly. The coefficient of determination (R^2) was used for heat map in this figure, including WT cells (**A**) and Δ*stm1* (**B**)
