# Supplementary material for: Implication of Stm1 in the protection of eIF5A, eEF2 and tRNA through dormant ribosomes
Source: Front Mol Biosci. 2024 Apr 18;11:1395220. doi: 10.3389/fmolb.2024.1395220 (PMC11063288; doi:10.3389/fmolb.2024.1395220)
Supplement: Supplementary file 1 [file DataSheet1.zip › Figure S9_new.pdf]

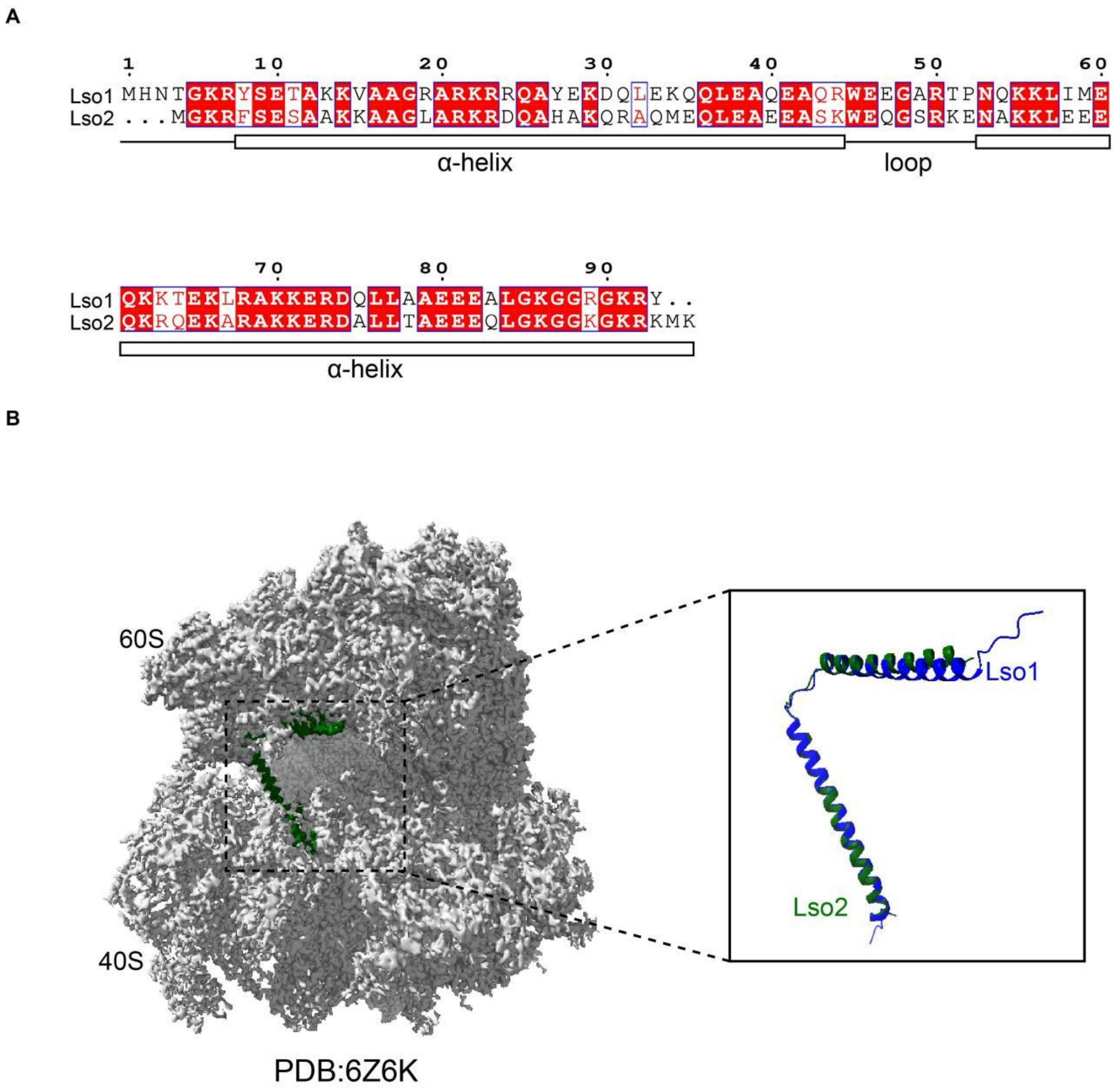

Figure S9. Lso1 is conserved to its homologue Lso2. A. Sequence comparison between Lso1 and Lso2. **B.** Structure of Lso2 on the dormant ribosome (PDB:6Z6K) and comparison between Lso1 (blue, AF-Q3E827-F1) and Lso2 (green).
