# Supplementary material for: Implication of Stm1 in the protection of eIF5A, eEF2 and tRNA through dormant ribosomes
Source: Front Mol Biosci. 2024 Apr 18;11:1395220. doi: 10.3389/fmolb.2024.1395220 (PMC11063288; doi:10.3389/fmolb.2024.1395220)
Supplement: Supplementary file 1 [file DataSheet1.zip › Table S1_new.pdf]

Table S1. Statistics of the cryoEM reconstruction and models

	80S •SERBP1 •eEF2 •eIF5A	80S •SERBP1 •eEF2 •E-tRNA	80S •Stm1 •eIF5A	80S •Stm1	80S •Stm1 •E-tRNA
**Source**	Human	Human	Yeast	Yeast	Yeast
**EMDB ID**	EMD-3799	EMD-37992	EMD-37995	EMD-37993	EMD-37994
**PDB ID**	8Y0W	8Y0X	8Y0U	N/A	N/A
**Data Collection**					
Particles for final reconstruction	73,781	85,882	9,646	447,912	135,726
Pixel size (Å)			1.08		
Defocus range (μm)			-0.6 to -3.5		
Voltage (kV)			300		
Electron dose ($e^{-}Å^{-2}$)			30		
**Model composition**					
Non-hydrogen atoms	220,490	218,907	196,274		
Protein residues	12,323	11,914	10,628		
RNA bases	5677	5753	5271		
Ligands (Zn^{2+}/Mg^{2+})	5/226	7/255	7/281		
**Refinement**					
Resolution (Å)	3.1	3.1	3.6		
$FSC_{average}$	0.89	0.90	0.86		
**Rms deviation**					
Bond lengths (Å)	0.008	0.006	0.007		
Bond angles (°)	0.772	0.723	0.808		
**Validation (proteins)**					
Molprobity score	2.12	2.05	2.36		
Clashscore, all atoms	13.27	11.22	20.09		
Good rotamers (%)	99.85	99.99	99.94		
**Ramachandran plot**					
Favored (%)	91.88	92.09	89.66		
Outliers (%)	0.10	0.08	0.21		
