# Supplementary material for: Implication of Stm1 in the protection of eIF5A, eEF2 and tRNA through dormant ribosomes
Source: Front Mol Biosci. 2024 Apr 18;11:1395220. doi: 10.3389/fmolb.2024.1395220 (PMC11063288; doi:10.3389/fmolb.2024.1395220)
Supplement: Supplementary file 1 [file DataSheet1.zip › Table S2.pdf]

Table S2. Data source of the mRNA-seq and Ribo-seq.

Sample	Description	Data type	Number of raw reads	Total base (bp)	Number of reads without adapter	Base of reads without adapter (bp)	Not aligned to non-conding RNA	Aligned to R64 genome	Ratio of available data
SRR16220048	rna_stm1_4_b7_s1	mRNA-seq	5,735,113	434,429,837	5,735,113	434,429,406	5,720,115	5,642,607	98.64%
SRR16220050	rna_stm1_3_b7_s1	mRNA-seq	6,093,696	455,622,520	6,093,696	455,622,033	6,076,369	5,987,990	98.55%
SRR16220052	rna_stm1_2_b7_s1	mRNA-seq	6,401,086	478,617,516	6,401,086	478,617,090	6,381,346	6,288,987	98.55%
SRR16220054	rna_stm1_1_b6_s1	mRNA-seq	5,997,290	448,442,427	5,997,290	448,442,075	5,980,918	5,900,698	98.66%
SRR16220069	rna_wt_4_b4_s1	mRNA-seq	7,081,275	529,456,596	7,081,275	529,456,186	7,061,994	5,844,925	98.57%
SRR16220070	rna_wt_3_b3_s1	mRNA-seq	5,278,623	394,683,691	5,278,623	394,683,326	5,263,315	5,207,573	98.94%
SRR16220071	rna_wt_2_b2_s1	mRNA-seq	7,843,356	586,374,743	7,843,356	586,374,250	7,821,031	7,729,722	98.83%
SRR16220072	rna_wt_1_b1_s1	mRNA-seq	5,735,978	428,918,966	5,735,978	428,918,640	5,719,723	5,648,984	98.76%
SRR16220044	ribo_wt_2_b2_s1	ribo-seq	33,695,760	2,544,250,324	33,695,760	1,242,604,721	33,661,329	7,425,638	22.06%
SRR16220046	ribo_wt_1_b1_s1	ribo-seq	32,173,389	2,433,696,212	32,173,389	1,181,816,086	32,151,583	8,026,262	24.96%
SRR16220073	ribo_stm1_4_b7_s1	ribo-seq	25,442,424	1,923,553,101	25,442,424	913,568,140	25,396,826	5,411,836	18.98%
SRR16220074	ribo_stm1_3_b7_s1	ribo-seq	28,262,224	2,137,327,932	28,262,224	944,818,844	28,224,776	4,174,274	14.79%
SRR16220075	ribo_stm1_2_b7_s1	ribo-seq	30,045,497	2,272,395,601	30,045,497	1,099,573,763	30,017,746	6,277,119	20.91%
SRR16220076	ribo_stm1_1_b6_s1	ribo-seq	33,343,704	2,521,680,293	33,343,704	1,256,498,690	33,324,342	8,736,950	26.22%
SRR16220085	ribo_wt_4_b4_s1	ribo-seq	34,206,425	2,585,754,721	34,206,425	1,234,150,264	34,183,732	7,862,562	22.83%
SRR16220086	ribo_wt_3_b3_s1	ribo-seq	39,398,314	2,979,491,611	39,398,314	1,385,667,637	39,349,860	7,802,584	19.83%
