# Supplementary figures and images for: Implication of Stm1 in the protection of eIF5A, eEF2 and tRNA through dormant ribosomes
Source: Front Mol Biosci. 2024 Apr 18;11:1395220. doi: 10.3389/fmolb.2024.1395220 (PMC11063288; doi:10.3389/fmolb.2024.1395220)

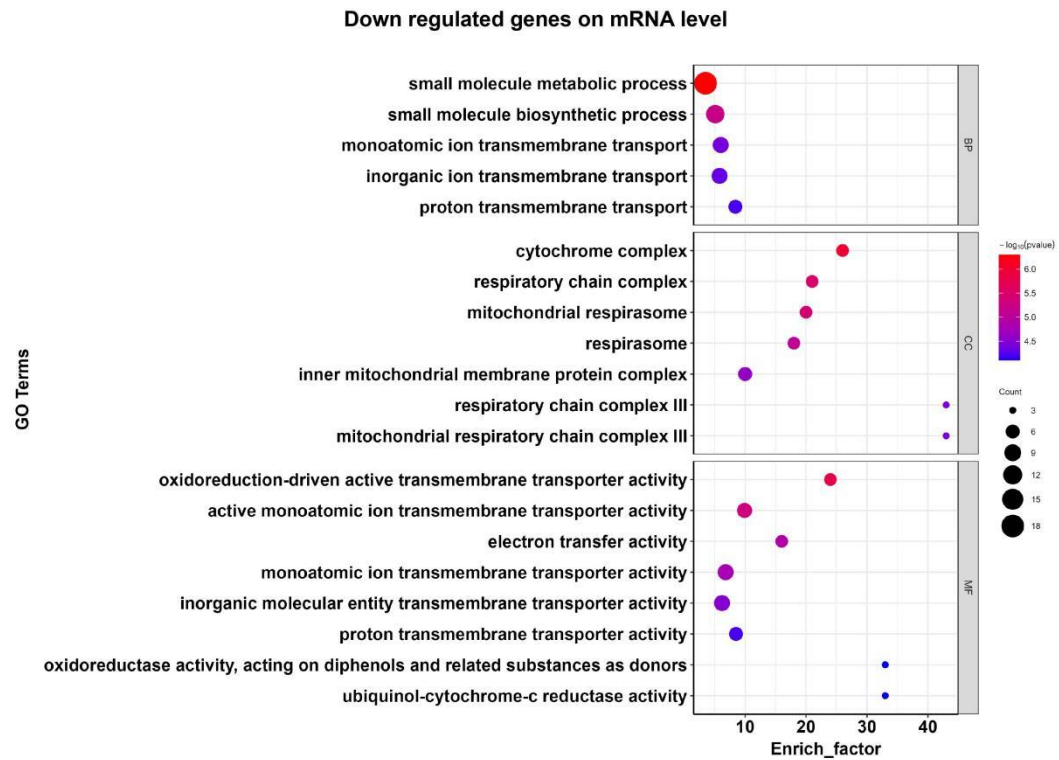

**Figure S10. GO pathway enrichment analysis of the 42 down regulated genes on transcriptional level.**

Supplement: Supplementary file 1 [file DataSheet1.zip › Figure S10_new.pdf]

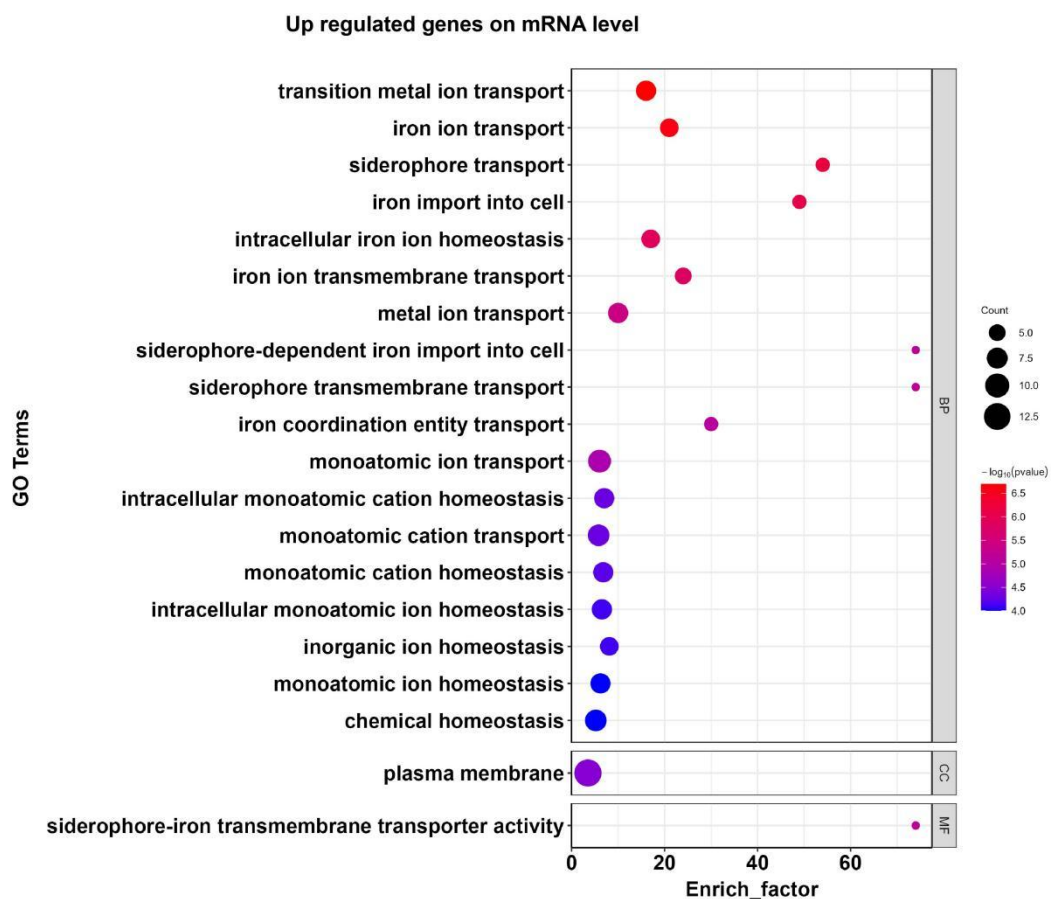

Figure S11. GO pathway enrichment analysis of the 62 up regulated genes on transcriptional level.

Supplement: Supplementary file 1 [file DataSheet1.zip › Figure S11_new.pdf]

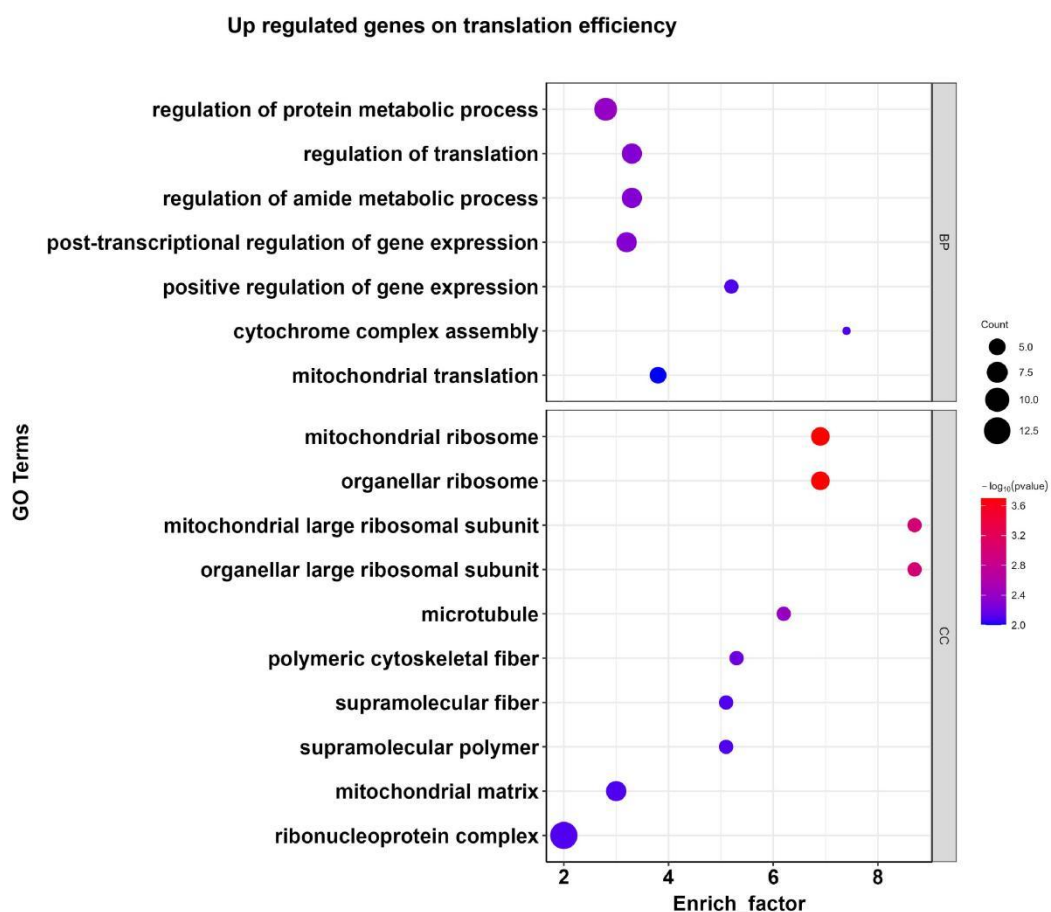

**Figure S12.** GO pathway enrichment analysis of the 63 up regulated genes on translation efficiency.

Supplement: Supplementary file 1 [file DataSheet1.zip › Figure S12_new.pdf]
